# X-Mapper: fast and accurate sequence alignment via gapped x-mers

**DOI:** 10.1186/s13059-024-03473-7

**Published:** 2025-01-22

**Authors:** Jeffry M. Gaston, Eric J. Alm, An-Ni Zhang

**Affiliations:** 1 Google, Cambridge, MA USA; 2 Department of Biological Engineering, Massachusetts Institute of Technology, Cambridge, MA USA; 3 School of Biological Sciences, Nanyang Technological University, Singapore, Singapore

**Keywords:** Bioinformatics, Sequence alignment algorithms, K-mer, Microbial sequencing

## Abstract

Sequence alignment is foundational to many bioinformatic analyses. Many aligners start by splitting sequences into contiguous, fixed-length seeds, called k-mers. Alignment is faster with longer, unique seeds, but more accurate with shorter seeds avoiding mutations. Here, we introduce X-Mapper, aiming to offer high speed and accuracy via dynamic-length seeds containing gaps, called gapped x-mers. We observe 11–24-fold fewer suboptimal alignments analyzing a human reference and 3–579-fold lower inconsistency across bacterial references than other aligners, improving on 53% and 30% of reads aligned to non-target strains and species, respectively. Other seed-based analysis algorithms might benefit from gapped x-mers too.

**Supplementary Information:**

The online version contains supplementary material available at 10.1186/s13059-024-03473-7.

## Background

Shotgun sequencing is applied broadly, such as in metagenomic studies of microbiomes [1], and data analysis requires researchers to process huge datasets and handle vast genomic diversity. Among all steps in processing shotgun sequences, read mapping is one of the most computationally intensive ones, and the quality of read mapping directly impacts the efficiency and accuracy of interpreting the sequencing data. Thus, this data challenge in sequencing-based biological studies calls for a sequence aligner with high accuracy, flexibility, and speed. Most sequence aligners have based their search algorithms on splitting sequences into seed sub-sequences of a fixed length, k (k-mers), and finding existing occurrences of those k-mers in a reference database, including Minimap [2] for read alignment, Blast [3] for similarity search, and Kraken [4, 5] for metagenomic taxonomy annotation. The use of k-mers in sequence alignment has made it possible to process large sequencing datasets efficiently and has enabled sequencing-based biological fields, including modern molecular biology and modern evolutionary biology.

A known challenge in k-mer algorithms is the requirement to choose a k-mer size [6], and the choice of k-mer size strongly affects sequence alignment — because any fixed k-mer size can be simultaneously too long to match genomic regions with high densities of mutations and too short to differentiate duplicated genomic regions. For a region with high mutational density, e.g., 1 mutation per 20 base pairs (bp), a long k-mer (e.g., 30 bp) would be unable to find a match. Meanwhile, for duplicated regions with more than 50 bp in length, a short k-mer (e.g., 10 bp) would find many matches. The genomic diversity among different species makes it clear that algorithms using fixed-size k-mers provide suboptimal flexibility for metagenomic studies.

**Fig. 1:**
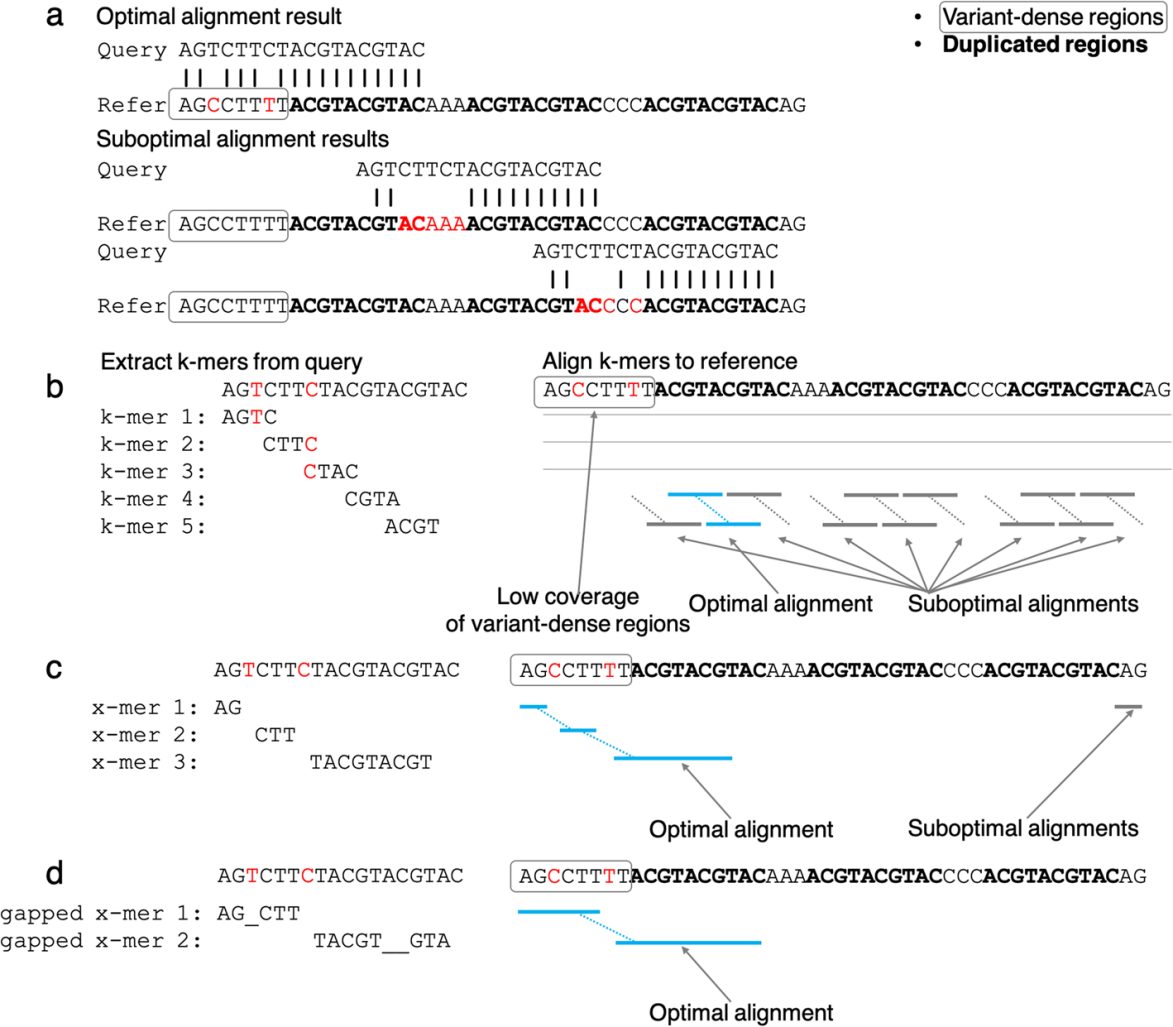
A toy example showing how an x-mer-based algorithm (**c**) can more effectively handle low-complexity repetitive regions and high-complexity variant-dense regions than a k-mer-based algorithm (**b**), and an algorithm using gapped x-mers (**d**) can further improve variant-dense regions. **a** The optimal alignment result and some candidate suboptimal alignment results of the example query mapping to the example reference. The reference is labeled as variant-dense regions and duplicated regions. **b**–**d** Matches of k-mers/x-mers/gapped x-mers highlighted in blue represent matches that agree with the optimal alignment. Matches of k-mers/x-mers/gapped x-mers highlighted in gray represent matches that do not agree with the optimal alignment but support suboptimal ones. The algorithm will then identify all unique offsets (positions of the query in the reference) to which any k-mer/x-mer/gapped x-mer maps. For each such offset, the algorithm may attempt to extend the k-mer/x-mer/gapped x-mer matches into neighboring base pairs to produce an alignment for the query at that position. Each dotted line connecting k-mers/x-mers/gapped x-mers represents a unique query offset where such an extension may occur. **b** K-mers 4 and 5 match each multiple offsets in the reference, while k-mers 1–3 have no matches. **c** X-mers 1–3 each match 1–2 offsets in the reference. **d** Gapped x-mers 1–2 match a single offset in the reference. Each “_” in a gapped x-mer represents a 1 bp gap

This conundrum is summarized in Fig. 1a. The k-mers overlapping mutations in the query sequence (common in variant-dense regions) — specifically k-mers 1–3 (Fig. 1b) — do not match anywhere in the reference genome; these k-mers are too long to avoid mismatches. In contrast, k-mers generated from duplicated regions in the query sequence — such as k-mers 4 and 5 (Fig. 1b) — match many locations in the reference genome. As a result, only 2 of the 12 matches (highlighted in blue) contribute to the optimal alignment (Fig. 1a), which is less efficiently found with this short k-mer size (gray matches in Fig. 1b). Therefore, for regions with mutations, a shorter k-mer size would be suitable to avoid mismatches, while for duplicated regions, a longer k-mer size would be more efficient to distinguish between those regions. Since both regions with mutations and duplicated regions exist within one genome, a fixed size of k-mers cannot simultaneously be optimal for all genomic regions. Although existing aligners allow customization of the k-mer size to tackle this problem for different genomes, even the most optimized k-mer size cannot solve this problem, since all k-mer-based aligners use a fixed size within a run.

An approach to address these limitations is to incorporate k-mers of varying sizes, which we call x-mers. The x-mer approach shortens the size of the x-mers at regions with mutations in the query sequence, allowing matches around point mutations (x-mers 1–2 in Fig. 1c) by avoiding issues encountered by k-mers 1–3 (Fig. 1b). For duplicated regions in the query sequence, we extend the x-mer size to distinguish between duplicated regions (x-mer 3 in Fig. 1c). In this example, all x-mers match 1–2 locations in the reference, and 3 of the 4 matches (highlighted in blue) contribute to the optimal alignment (Fig. 1a), compared to 2 of the 12 matches found using k-mers (Fig. 1b). Moreover, x-mer matches at the optimal offset can cover 6 of 8 bp of the variant-dense region, while k-mer matches at the optimal offset show no coverage of the variant-dense region. These results suggest that x-mers can describe the appropriate alignment more precisely and utilize more information in the query. However, short x-mers that adapt to dense point mutations (e.g., x-mers 1 and 2) are more likely to align to multiple suboptimal offsets, reducing their specificity.

While variable-length seeds (x-mers) have previously been used by LAST [7] for local alignment, and spaced seeds were first introduced in PatternHunter [8] and later widely adopted by many aligners like SHRiMP2 [9], we introduce the concept of dynamically incorporating k-mers of dynamic sizes and gaps, which we call gapped x-mers. We believe the most interesting contribution of the gapped x-mer is its ability to offer both variable length and gaps at the same time. This can be done without storing every such possible seed by carefully selecting which ones to store, in a manner reminiscent of minimizers [2]. Instead of necessarily shortening the size of k-mers in variant-dense regions, this algorithm generates gaps that can skip over different combinations of mutations (as illustrated by gapped x-mer 1 in Fig. 1d), allowing them to remain more specific. As a result, each gapped x-mer in this example matches exactly one location in the reference, identifying the optimal alignment within a single search. This improvement in precision, specificity, and coverage allows gapped x-mers to align more effectively and quickly.

We applied the gapped x-mer concept to short-read alignment and developed a short-read aligner, X-Mapper (https://github.com/mathjeff/Mapper [10]). Overall, X-Mapper is a highly accurate tool for sequence alignment, and it reduces the number of suboptimal alignments compared to other alignment tools. X-Mapper has been tested in diverse microbiome sequencing samples including whole genome sequencing data and metagenomes, because we expected gapped x-mer-based algorithms to accommodate diverse combinations of regions with mutations and duplicated regions in different species better than k-mer-based algorithms. The high accuracy, flexibility, and speed of X-Mapper can benefit diverse biological studies that rely on shotgun sequencing. X-Mapper may also help with larger genomes such as human genomes after further refinement.

We envision that the new concept of the gapped x-mer algorithm can inspire new seeding and can help improve other k-mer-based and x-mer-based bioinformatic applications in the future, such as similarity search (BLAST [3], Diamond [11]), taxonomy assignment in metagenomes (Kraken [4, 5]), multiple sequence alignment (MUSCLE [12], MAFFT [13]), and genome assembly (SPAdes [14], IDBA [15]).

## Results

### X-Mapper algorithm

**Fig. 2:**
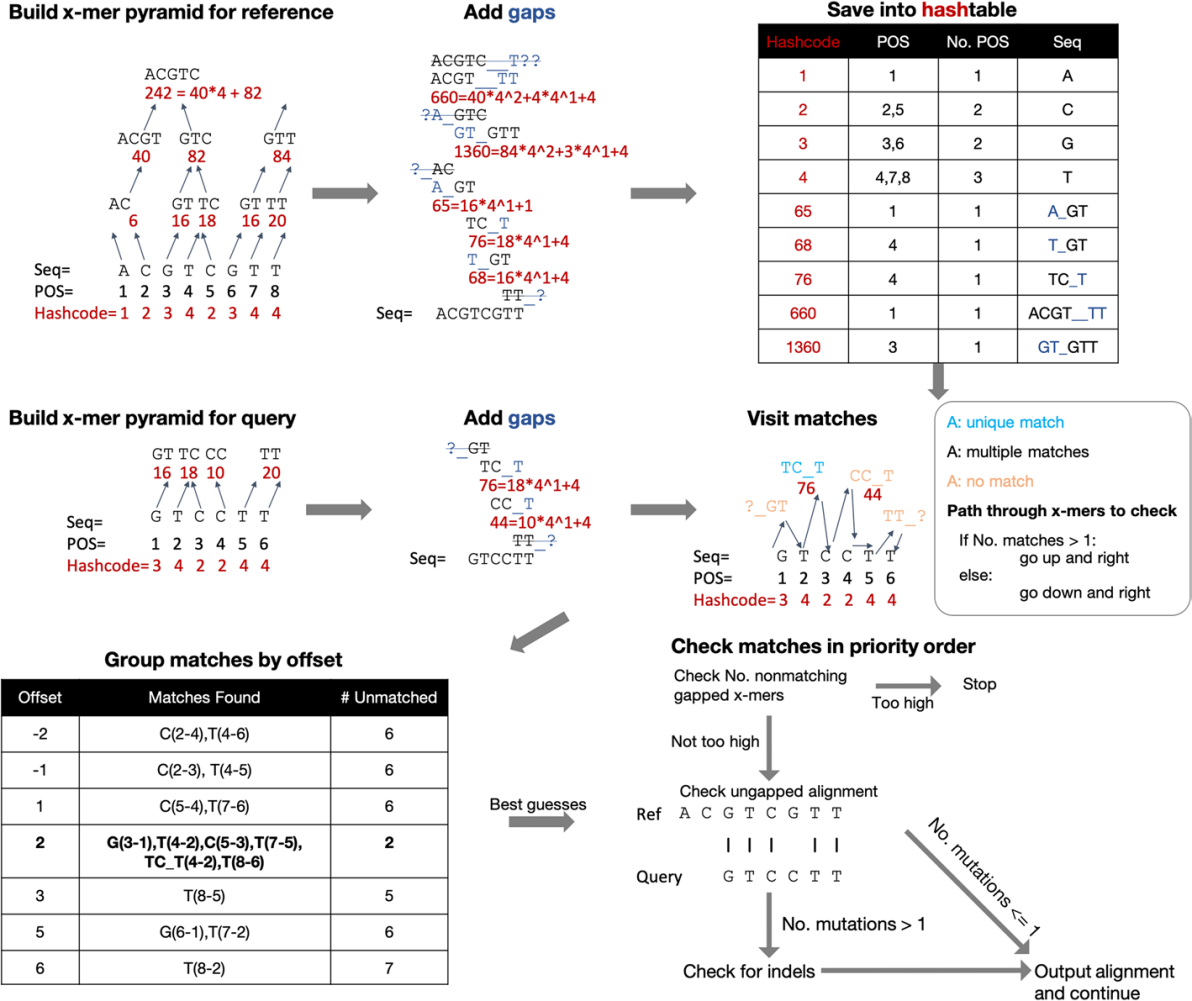
X-Mapper’s algorithm. X-Mapper starts by building a pyramid of x-mers. In this simplified example, larger x-mers are built by merging smaller x-mers left if number of A’s plus C’s is odd, and right if the number of A’s plus C’s is even. The hashing function of the leaf node is A = 1, C = 2, G = 3, T = 4. The hashing function for a larger child x-mer, in this simplified example, is 4* the hashcode of the left parent x-mer + the hashcode of the right parent x-mer. After building x-mers, we expand most x-mers into gapped x-mers. In this simplified example, x-mers choose to extend their gaps to the left if their number of A’s plus G’s is odd; otherwise, they choose to extend their gaps to the right. Gapped x-mers that are excessively short, and expected to match to too many places (about 16) in a random reference genome, are not generated. Each resulting gapped x-mer is assigned a hashcode and saved into a hashtable for fast lookup. Hashcodes are highlighted in red. The hashtable contains the hashcode and positions (POS) of all gapped x-mers built from the reference. The sequences of the gapped x-mers are only shown here for clarity. While walking through an x-mer pyramid of the query, X-Mapper searches for possible offsets (position of the query in the reference) for the optimal alignment by expanding each x-mer into a gapped x-mer and looking for matches of the gapped x-mer in the reference. If the number of matches is large compared to the number of base pairs specified in the gapped x-mer, the path advances up and right, increasing the number of base pairs used by the following seed. If the number of matches is small, the path advances down and right, removing some base pairs from the following seed. While following this path through the gapped x-mer pyramid, X-Mapper groups matches of interesting gapped x-mers to determine which offset in the reference to check first (referred to as the optimistic best offset). If the penalty of an ungapped alignment is no more than an indel, X-Mapper considers the result to be the final alignment for this offset. If the penalty of the initial ungapped alignment is at least that of one indel, X-Mapper checks for indels first. If there may be other co-optimal or superior alignments, X-Mapper continues to look for all of them before outputting any of them

In this study, we designed a gapped x-mer-based algorithm that uses gapped x-mers of all possible sizes and used this algorithm to develop a new short-read alignment tool, X-Mapper. X-Mapper starts by building a pyramid of x-mers from 1 base pair up to the entire length of the sequence if needed (Fig. 2, details in “Methods”). After building x-mers, X-Mapper generates gapped x-mers that we expect to be long-enough based on the length of the reference, by adding an additional x base pairs plus a pseudorandom number of base pairs from 0 through 2 (specifically the hashcode modulo 3) to avoid skipping generating gapped x-mers using certain numbers of base pairs. These approximately x base pairs are evenly split into gap and extension base pairs, resulting in a gap of approximately x/2 base pairs and an extension of approximately x/2 base pairs. Each x-mer chooses its gap direction based on the contents of the x-mer itself. Then, X-Mapper generates a hashcode for each gapped x-mer. Gapped x-mers from the reference are then saved to a hashtable. These gapped x-mers are generated for the reference genome and each query sequence using the same algorithm to enable finding matches in the hashtable. When generating the x-mer pyramid, X-Mapper shifts x-mers by roughly one quarter of their length each time (instead of generating all x-mers and storing their resulting gapped x-mers), which allows indexing the sequences more quickly with less memory. This pyramid structure facilitates efficient, dynamic selection of an appropriate gapped x-mer for a specific section of a query sequence based on the distribution of mutations and homology. As a result, the users will not accidentally use a wrong k-mer size, e.g., 19–22 mers for a microbe genome (default of some existing aligners).

With both x-mer pyramids of the query and the reference, X-Mapper searches for potential offsets (positions of the query in the reference) for the optimal alignment with as many and as long gapped x-mer matches as possible. To do that, X-Mapper starts from the bottom of the pyramid, i.e., the first x-mer of length 1 base pair, and moves up and down the pyramid based on the number of reference genomic positions the corresponding gapped x-mer aligns to (matches). For gapped x-mers that match too many duplicated genomic regions, X-Mapper proceeds up and right in the pyramid to check longer x-mers to distinguish among different genomic regions, and for gapped x-mers yielding too few due to overlapping regions with mutations, X-Mapper proceeds down and right in the pyramid to check x-mers of shorter sizes (details in “Methods”) (Fig. 2). In this way, X-Mapper efficiently finds interesting x-mers to check instead of iterating through all x-mers in the pyramid.

To identify potential optimal alignments, X-Mapper first optimistically searches for the best alignment by following this x-mer path until one reference offset is found having more x-mer matches than any other, and at least two query x-mers match to a close reference offset. If this process yields exactly one offset, X-Mapper checks it first and attempts to determine (using the number of nonoverlapping x-mers that do not match at other offsets) that it is the best alignment. If X-Mapper is unable to determine that this optimistic alignment is optimal, then X-Mapper continues to follow this x-mer path. Whenever a reference new offset is discovered having at least 2 x-mer matches, X-Mapper attempts an alignment at this offset. If the x-mer path reaches the end of the query, or X-Mapper can demonstrate that no better alignment can exist, this search terminates.

Most of the time, X-Mapper does not need to attempt alignments at offsets other than the optimal one. In the toy example, a k-mer-based aligner would require checking eight additional offsets (gray dotted lines) to identify the best alignment (Fig. 1a), which could be missed if sufficient time is not allotted. Using gapped x-mers, we can find the best match after checking a single offset (Fig. 1d).

### Alignment accuracy of X-Mapper in samples with various complexities

**Fig. 3:**
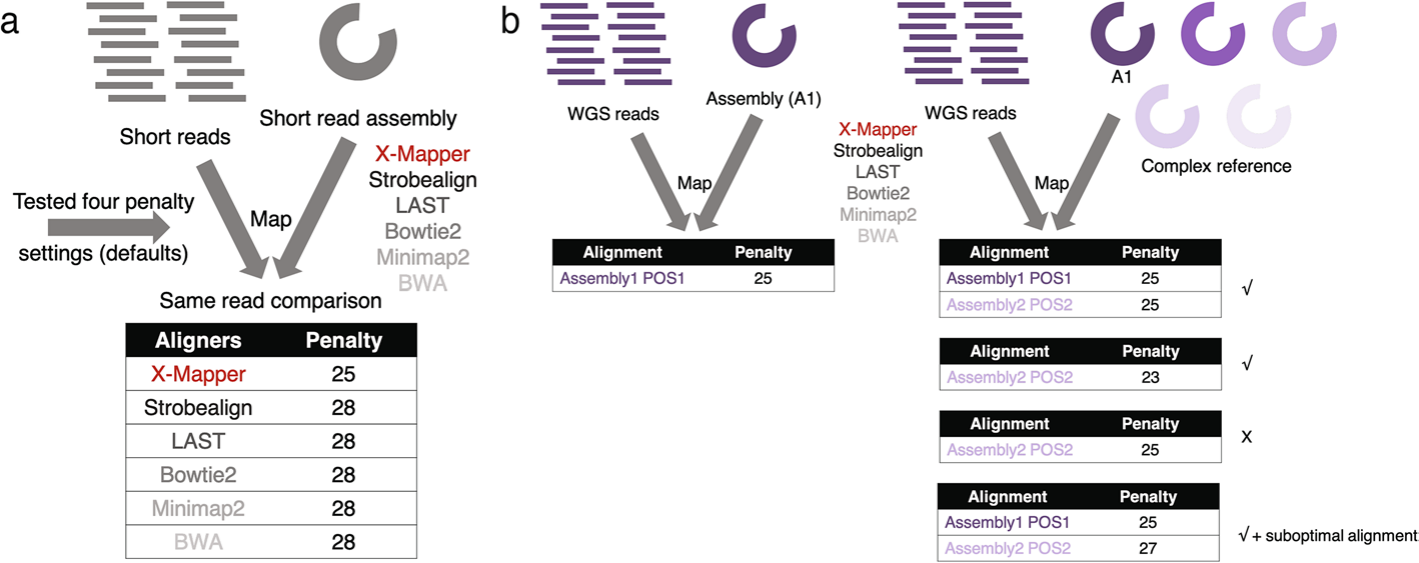
Evaluating the alignment accuracy and consistency of X-Mapper in sequencing samples of various complexities. Penalties shown are example penalties for a single read. **a** Alignment accuracy was evaluated by comparing the alignment results of the same reads to the same reference genome under the same penalty (alignment score) settings across different aligners. Alignments with the lowest penalty (higher alignment score) were considered optimal, while all other alignments with higher penalties (lower alignment scores) were declared suboptimal. **b** Alignment consistency was evaluated by comparing the alignment results of the same reads by the same aligner in one reference (Assembly A1) versus a complex reference (multiple genomes). Alignments were considered consistent if (1) the optimal alignments to a simple reference were also reported when aligned to a complex reference, or (2) a complex reference yielded better alignments (with lower penalties or higher alignment scores) than the simple reference, primarily due to assembly errors. The alignment consistency was then compared across aligners

To investigate the alignment accuracy of X-Mapper, we first tested alignment penalties (alignment scores) from X-Mapper against those of k-mer-based algorithms Strobealign and Minimap2, and x-mer-based methods Bowtie2, BWA (referring to BWA MEM in this study), and LAST. Since each aligner uses a different penalty formula for spacing, appropriately comparing penalties of paired-end alignment results across aligners is not well defined. Thus, for this evaluation we tested single-read alignment by comparing the results from different aligners for the same read. Specifically, we examined how well each aligner aligned the same reads to the same reference genome under the same penalty settings (Fig. 3a). We tested two scenarios: a human gut microbiome metagenome [16] mapping to its own assembly and a human transcriptomic dataset mapping to a complete human genome reference. Four penalty settings were tested, including the default settings of X-Mapper, Bowtie2, Minimap2, and BWA. We compared the alignment results of the same reads reported by the four aligners and identified the alignments with the lowest penalty (highest alignment score) as optimal. Here, we used a stand-alone script to re-compute the penalty of an alignment, using the CIGAR string and position reported by each aligner which determined the number of matches, mismatches, ambiguous matches, gap opens, and gap extensions. Alignments with higher penalties (lower alignment scores) were declared suboptimal. We then calculated the percentage of reads reported with suboptimal alignments for each aligner.

**Fig. 4:**
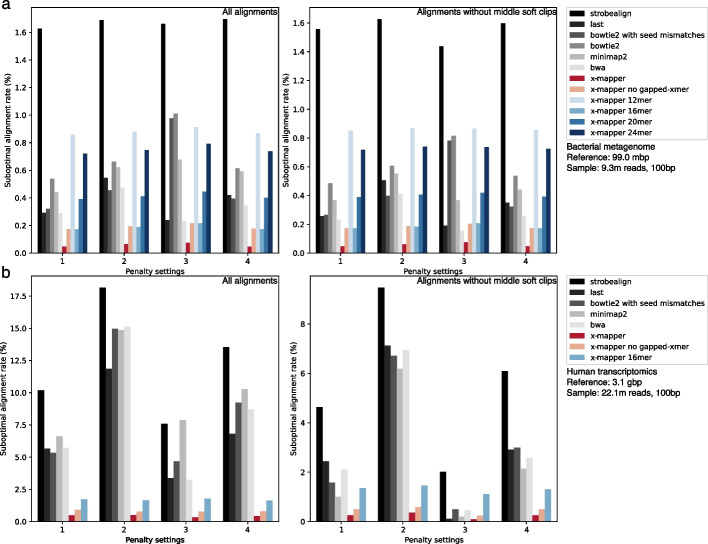
X-Mapper exhibits higher alignment accuracy with a lower suboptimal alignment rate (percentage of reads reported with suboptimal alignments) than other aligners, when applied to **a** a bacterial metagenome and **b** a human transcriptome. Optimal alignments were identified as those with the lowest penalty (highest alignment scores) reported by all aligners. Each sample was tested using four different penalty settings, including the default settings of (1) Bowtie2, (2) BWA (referring to BWA MEM in this study), (3) X-Mapper, and (4) Minimap2 (x-axis). Reads where local aligners, such as Minimap2, reported soft clips in the middle of the contigs were removed from downstream analysis (“alignments without middle soft clips”)

Local aligners like Minimap2 and BWA often report soft clips in the middle of reference contigs. For short-read alignment, we consider soft clips to be less significant. Both X-Mapper and Bowtie2 are end-to-end aligners that prioritize alignment contiguity. LAST can be configured for end-to-end alignment using the “-T 1” option. BWA and Minimap2 can achieve this by adjusting soft clip penalties with the “-L” and “-z” options, respectively. We also configured Minimap2 with the “-ax sr” setting for short reads. However, adjusting the soft clip penalty (“-z”) in Minimap2 did not fully prevent soft clip reporting. Thus, for a more interesting comparison, we analyzed the alignment results of all reads and alignment results of reads without middle soft clips reported by Minimap2 (Fig. 4 “alignments without middle soft clips”).

Our results demonstrate that X-Mapper exhibits higher alignment accuracy than all other aligners, indicated by a lower suboptimal alignment rate (Fig. 4). Specifically, when aligning a human gut microbiome metagenome (Fig. 4a “all alignments”), X-Mapper shows a suboptimal alignment rate of 0.05% out of 9.3 million reads, which is 6–34 times lower than those of Strobealign (1.63%), LAST (0.29%), Bowtie2 (0.53%), Minimap2 (0.44%), and BWA (0.29%) under penalty setting #1. After excluding alignments where Minimap2 reported middle soft clips (Fig. 4a “alignments without middle soft clips”), X-Mapper maintains a suboptimal alignment rate of 0.05%, which is 5–33 times lower than those of Strobealign (1.55%), LAST (0.26%), Bowtie2 (0.48%), Minimap2 (0.37%), and BWA (0.23%). Configuring Bowtie2 to allow seed mismatches (“-N 1”) improves its accuracy from 0.48 to 0.27%, a trend observed across all penalty settings. Therefore, we used this Bowtie2 configuration with seed mismatches for downstream analysis. For Strobealign, we tested it with both default penalties and adjusted penalties relative to different alignment scores (details in “Methods”). However, it still exhibited a relatively high suboptimal alignment rate, with the lowest being 0.96%.

When aligning a human transcriptomic dataset to a complete human genome reference, we found that X-Mapper demonstrates a suboptimal alignment rate of 0.48%, which is 11–24 times lower than those of Strobealign (10.2%), LAST (5.7%), Bowtie2 with seed mismatches (5.3%), Minimap2 (6.6%), and BWA (5.7%) under penalty setting #1 (Fig. 4b “all alignments”). After removing alignments where Minimap2 reported middle soft clips, X-Mapper maintains a suboptimal alignment rate of 0.25%, which is 4–18 times lower than those of Strobealign (4.6%), LAST (2.4%), Bowtie2 with seed mismatches (1.58%), Minimap2 (1.0%), and BWA (2.1%) (Fig. 4b “alignments without middle soft clips”).

To investigate X-Mapper’s performance with more divergent references (e.g., not the same bacterial strain), we used whole genome sequencing (WGS) data from *Bacteroides fragilis* aligning to its own assembly, to reference genomes representing different strains of the same species, and to reference genomes representing other *Bacteroides* species, covering ANI values [17] from 78.7 to 100.0%. X-Mapper demonstrated the highest accuracy with the lowest suboptimal alignment rates compared to other aligners, a trend consistent across all ANI values and penalty settings (Additional file 1: Fig. S1). For instance, when aligning *B. fragilis* reads to a *B. xylanisolvens* reference genome (ANI = 78.7%), X-Mapper shows 6–53-fold lower suboptimal alignment rates than other aligners (under penalty setting #1). When aligning *B. fragilis* reads to a different *B. fragilis* strain isolated from the same person (ANI = 99.99%), X-Mapper shows 58–415-fold lower suboptimal alignment rates than other aligners (under penalty setting #1).

### What makes X-Mapper more accurate?

To determine the factors contributing to this high alignment accuracy, we built modified versions of X-Mapper that use fixed k-mer sizes or x-mers with no gaps. We found that the ability to use dynamic k-mer sizes contributes significantly to the high accuracy of X-Mapper. Specifically, X-Mapper with ungapped x-mers showed a 4-fold higher suboptimal alignment rate (0.17%) compared to X-Mapper with gapped x-mers (0.05%). In addition, X-Mapper with a fixed 12-mer size showed the worst performance, with a suboptimal alignment rate of 0.85% under penalty setting #1 (Fig. 4a “alignments without middle soft clips”). Increasing the k-mer size from 12 to 16 bp greatly improved accuracy, while further increasing the k-mer size from 16-mer to 24-mer decreased accuracy from 0.17 to 0.72%. The k-mer sizes tested in our study cover the default minimum k-mer sizes used by Strobealign (20-mer), Bowtie2 (22-mer), BWA (19-mer), and Minimap2 (21-mer).

In addition, the suboptimal alignment rates reported by X-Mapper with ungapped x-mers and fixed-size k-mers were comparable to the suboptimal rates of all the other aligners, except Strobealign. Based on this observation, we hypothesized that the absence of gaps and suboptimal k-mer sizes are likely the primary reason for suboptimal alignments in other aligners.

**Fig. 5:**
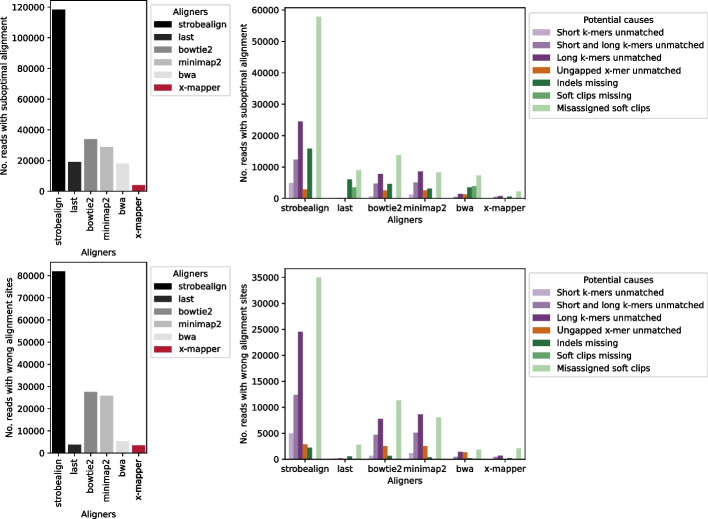
Potential causes of suboptimal alignments when aligning a gut microbiome metagenome, after excluding alignments with middle soft clips reported by Minimap2. Suboptimal alignments were classified into four types: reads failed to align, aligned to the wrong sites, aligned to close sites (within 100 bp), or aligned to the same sites but with a higher (suboptimal) penalty score compared to the optimal alignments. Reads that failed to align or aligned to the wrong sites were considered more serious suboptimal alignment types due to their greater potential to impact downstream analysis. The Bowtie2 configuration allowing 1 seed mismatch was tested in this analysis

To test this hypothesis, we explored the potential causes of suboptimal alignments by comparing the alignment results of a single read across different aligners and X-Mapper with fixed k-mer sizes. We grouped the suboptimal alignments into different types: reads that failed to align or aligned to the wrong sites, close sites (within 100 bp), or the same sites but with a higher (suboptimal) penalty score compared to the optimal alignments. Reads that failed to align or aligned to the wrong sites were considered more serious suboptimal alignments due to their greater potential to impact the downstream analysis. When aligned to a bacterial metagenome, these wrong alignment sites accounted for 69.2% (Strobealign), 19.9% (LAST), 81.2% (Bowtie2), 89.5% (Minimap2), 30.1% (BWA), and 86.9% (X-Mapper) (Fig. 5). The other two types of suboptimal alignments included reads aligned to close sites (within 100 bp) or the same sites but with a higher (suboptimal) penalty score as the optimal alignments (details provided in the following section). These suboptimal alignments identified approximate sites in the reference but reported a different arrangement of matches, such as indels mis-assigned as point mutations or soft clips. These types of suboptimal alignments could also be critical for downstream analyses, such as genetic variant identification.

We then grouped alignment results by their potential causes. First, if X-Mapper with fixed k-mer sizes (12 mers, 16 mers, 20 mers, and 24 mers) could replicate a suboptimal alignment, suboptimal k-mer sizes (short k-mers unmatched, short and long k-mers unmatched, and long k-mers unmatched) were considered a potential cause (Additional file 1: Fig. S2). Specifically, if X-Mapper 12 mers reported the optimal alignments for a read, but X-Mapper with a longer k-mer size (16–24 mers) reported suboptimal alignments with the same suboptimal score as the other aligner, we considered that unmatched long k-mers might be the potential cause of suboptimal alignments in the other aligners. Conversely, if X-Mapper 24 mers reported the optimal alignments, but X-Mapper with a shorter k-mer size (12–20 mers) reported suboptimal alignments with the same suboptimal score as the other aligner, we considered unmatched short k-mers as the potential cause. If X-Mapper 16 mers or 20 mers found the optimal alignments, but both 12 mers and 24 mers reported suboptimal alignments with the same suboptimal score as the other aligner, we considered that both unmatched short and long k-mers might be the main cause. In addition, if X-Mapper with ungapped x-mers could replicate a suboptimal alignment with the same suboptimal score as the other aligner, then ungapped x-mers were considered a potential cause.

For suboptimal alignments that X-Mapper with fixed k-mer sizes and ungapped x-mers could not explain, we examined the details of the alignment results (Additional file 1: Fig. S2). If a soft clip or an indel was only found in the optimal alignment, we considered not finding the optimal alignment containing a soft clip or an indel (i.e., missing soft clips or indels) as potential causes for suboptimal alignments. Additionally, if soft clips were reported in suboptimal alignments but not in the optimal alignment, we considered mis-assigned soft clips as the potential cause.

When aligning the gut microbiome metagenome dataset, ungapped x-mers and suboptimal k-mer sizes (short k-mer unmatched, long k-mer unmatched, or both) can be a primary cause of suboptimal alignments. For this analysis, alignments reported with middle soft clips by Minimap2 were excluded. Specifically, ungapped x-mers and suboptimal k-mer sizes account for 37.8% of suboptimally aligned reads in Strobealign (118,374 reads), 2.0% in LAST (18,983 reads), 46.0% in Bowtie2 (33,947 reads), 60.4% in Minimap2 (28,850 reads), 17.9% in BWA (17,872 reads), and 29.8% in X-Mapper (3978 reads) (Fig. 5). It is not surprising that LAST’s results are least explained by ungapped x-mers and suboptimal k-mer sizes, as its variable-length seeding approach closely resembling the x-mer algorithm of X-Mapper, which generates seeds long enough for specificity and short enough for sensitivity. The remaining suboptimal alignments were attributed to missed indels, missed soft clips, and mis-assigned soft clips.

Regarding wrong alignment sites, i.e., reads that failed to align or aligned to the wrong sites, we found that X-Mapper reported the fewest compared to the other aligners: 3458 reads, compared to 81,880 (Strobealign), 3783 (LAST), 27,573 (Bowtie2), 25,823 (Minimap2), and 5386 reads (BWA) (Fig. 5). Specifically, ungapped x-mers and suboptimal k-mer sizes can explain almost half of the wrong alignment sites for Strobealign (54.6%), Bowtie2 (56.6%), Minimap2 (67.5%), and BWA (59.2%) (Fig. 5). However, mis-assigned soft clips can explain 60.0% of wrong alignment sites for X-Mapper (Fig. 5). This suggests that inflexible k-mers and x-mers are one of the primary causes of wrong alignment offsets for the other aligners, while X-Mapper could improve on soft clip identification for more accurate alignments.

Here, we show an example of a read aligned to a reference offset with eight point mutations as the optimal alignment. We found that only X-Mapper and LAST were able to align this read, while the other aligners could not (Additional file 1: Fig. S3). We tested X-Mapper with ungapped x-mers and a series of fixed k-mer sizes (12 mers to 24 mers) and found that X-Mapper with 14 mers, 16 mers, 18 mers, and 20 mers were able to report the optimal alignment, but not X-Mapper with ungapped x-mers, 12 mers, 22 mers, or 24 mers. This case shows that both short and long k-mers can be unmatched during alignment. However, a shorter k-mer size appeared to work for Strobealign (14 mers aligned but not 15 mers), Bowtie2 (14 mers aligned but not 18 mers), Minimap2 (12 mers and 14 mers aligned but not 16 mers), and BWA (10 mers, 12 mers, 14 mers aligned but not 18 mers).

When aligning the human transcriptomic dataset, we found that the ability to use various k-mer sizes and gaps accounts for only a small proportion of the high accuracy of X-Mapper. Specifically, ungapped x-mers and suboptimal k-mer sizes could explain 7.3% (of 1,021,097 reads with suboptimal alignments for Strobealign), 7.2% (of 537,990 reads for LAST), 10.6% (of 348,828 reads for Bowtie2), 10.7% (of 221,094 reads for Minimap2), 9.7% (of 463,526 reads for BWA), and 0% (of 55,193 reads for X-Mapper) (Additional file 1: Fig. S4). Instead, missing indels and soft clips were the common causes of suboptimal alignments. However, for wrong alignment offsets, ungapped x-mers and suboptimal k-mer sizes were a more important cause, contributing to 34.8% (of 58,928 reads with wrong alignment sites for LAST), 32.7% (of 106,880 reads for Bowtie2), 25.9% (of 84,990 reads for Minimap2), and 22.6% (of 154,179 reads for BWA). For Strobealign and X-Mapper, mis-assigned soft clips can explain 71.2% of 640,952 reads with wrong alignment sites and 66.5% of 36,842 reads, respectively. These observations are consistent with the microbial metagenomic sample, suggesting that the ability of an alignment algorithm to use flexible k-mer sizes and gaps is crucial for achieving high accuracy in sequencing datasets.

### Suboptimal alignments aligning to close sites or the same sites

Sometimes, aligners identified approximate sites in the reference genome but reported different arrangements of matches, resulting in higher penalties or suboptimal alignments. For example, X-Mapper, Strobealign, Bowtie2, Minimap2, and BWA aligned the same read to nearby sites (POS 170,702–170,764) in the reference (Additional file 1: Fig. S5). Among these alignments, X-Mapper reported the lowest penalty of 66 with an insertion of 5 bp (in the query), two point mutations, another insertion of 2 bp, a deletion of 2 bp, and two additional point mutations. Bowtie2 reported the second-lowest penalty of 70, detecting one point mutation, an insertion of 9 bp (in the query), another insertion of 5 bp, and two more point mutations.

In contrast, Minimap2 and BWA did not compute potential indels and instead reported a soft clip of 25 bp. Soft clips located in the middle of contigs were treated as indels in our analysis. Strobealign aligned the read with a high penalty of 216, indicating 36 point mutations within 44 bp, which may be attributed to a potential bug in handling complex alignments. LAST did not report any alignment for this read. Since our downstream analysis excluded reads where Minimap2 reported middle soft clips, this read is included here only as a demonstration.

Another example shows aligners (LAST, Bowtie2, Minimap2, BWA, and X-Mapper) reporting alignments of one read to the same site in the reference (POS 24,316) while disagreeing on the arrangement of matches (Additional file 1: Fig. S6). In this case, Minimap2 reported the arrangement with the lowest penalty of 16, represented by one point mutation and a soft clip of 10 bp. However, Minimap2 discarded the last 5 bp in the reference as part of the soft clip, which should instead be considered as two point mutations. X-Mapper reported the second-lowest penalty of 23, identifying three point mutations and a 5 bp soft clip.

BWA reported a slightly higher penalty of 29, identifying one point mutation, a deletion of 1 bp (in the query), another deletion of 1 bp, and a soft clip of 7 bp. LAST reported a different arrangement involving two point mutations, an insertion of 4 bp (in the query), and another insertion of 1 bp, yielding a penalty of 37. Bowtie2 reported a slightly higher penalty of 38, identifying one point mutation and an insertion of 9 bp (in the query). Strobealign aligned the read to a nearby site in the reference (POS 24,265) with a high penalty of 380, identifying 80 point mutations. This high penalty may be caused by the same bug in Strobealign when handling complex alignments.

X-Mapper invests a substantial fraction of its effort in identifying a better arrangement of matches to achieve an alignment with the lowest total penalty. This can be done efficiently using the A* search algorithm [18], which searches the Needleman-Wunsch grid to determine the path with the minimum penalty. X-Mapper also actively prunes any branches that have exceeded the maximum indel extension length calculated previously for this alignment site. This thorough and efficient search for indels allows X-Mapper to find an optimal alignment without consuming significantly more time.

### Alignment consistency of X-Mapper in samples with various complexities

In addition to low alignment penalty, it is crucial for an aligner to report consistent alignments of the same pair of paired-end reads when aligned to a superset (representing diverse microbial species) of its original reference (a single bacterium isolate) (Fig. 3b). To test alignment consistency, we aligned a WGS dataset (3.2 million paired-end reads, 150 bp) of *B. fragilis* to its own assembly, representing the simple reference. We also aligned this WGS dataset to a genome collection of 88 human gut microbiome strains (441.4 Mbp in total), representing the complex reference. This collection included (1) the *B. fragilis* WGS assembly (Assembly1); (2) 5 other *B. fragilis* references representing different strains; (3) 55 *Bacteroides* species that are not *B. fragilis*; and (4) 27 species that are not *Bacteroides*. These 88 genomes competed for the WGS reads during the alignment. For each read, we compared its alignment results to the complex reference with those to the simple reference (true alignment site) reported by the same aligner (Fig. 3b). Then, the consistency of alignment was compared across aligners. We characterized an alignment as consistent if: (1) the optimal alignments to the simple reference were also reported when aligned to the complex reference, or (2) the complex reference found better alignments (with lower penalties or higher alignment scores) than those to the simple reference (mainly due to assembly errors). We then computed inconsistency as the percentage of reads that were not reported with consistent alignments to the complex reference compared to the simple reference.

We found that the default setting for Bowtie2, Minimap2, BWA, and Strobealign is to report a single alignment. Therefore, we also ran these aligners under a setting that reports multiple (i.e., 100), if not all, alignments. As a result, suboptimal alignments were often reported with this “all” setting enabled. While reporting suboptimal alignments is not technically wrong, it is inefficient for users because they need to filter the alignments before downstream analysis. Additionally, the secondary alignments can have 4–5 more point mutations compared to the optimal alignments, even when the optimal alignments have perfect matches. Reporting these suboptimal alignments seems unhelpful. Consequently, we also computed the percentage of reads reported with suboptimal alignments when aligned to the complex reference.

**Fig. 6:**
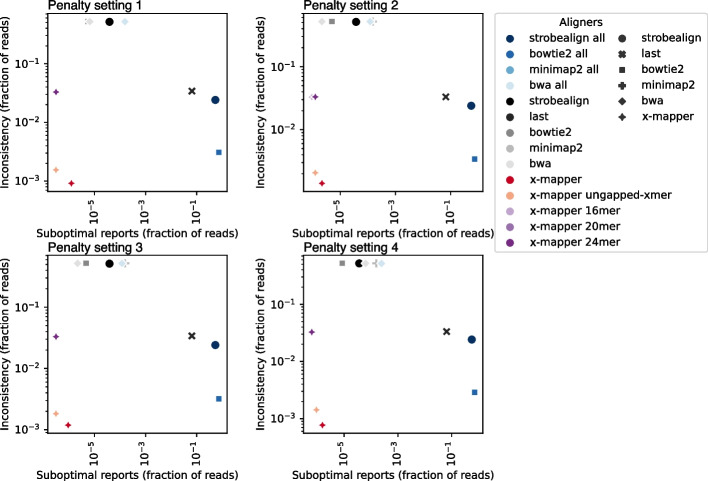
X-Mapper exhibits the lowest alignment inconsistency (fraction of reads reported with inconsistent alignments) and the fewest suboptimal alignments (fraction of reads reported with suboptimal alignments) when aligned to a complex reference. Alignment consistency was tested by comparing the alignment results of a whole genome sequencing (WGS) sample aligning to 88 bacterial genomes (representing a complex reference), to the alignment results of this WGS sample aligning to its own assembly (representing a simple reference). An alignment was considered consistent if: (1) the optimal alignments to the simple reference were also reported when aligned to the complex reference, or (2) the complex reference yielded better alignments (with lower penalties or higher alignment scores) than those in the simple reference. Bowtie2, Minimap2, and BWA were run under their default settings and settings that reports multiple, if not all, alignments (labeled as “all”). Since the “all” settings often report suboptimal alignments, the fraction of reads reported with suboptimal alignments was also evaluated for each aligner. Alignment consistency was tested using four different penalty settings, including the default settings of (1) Bowtie2, (2) BWA, (3) X-Mapper, and (4) Minimap2. The Bowtie2 configuration with seed mismatches was tested in this analysis

We found that X-Mapper reported the lowest inconsistency and fewest suboptimal alignments compared to other aligners. For example, under penalty setting #1, X-Mapper showed an inconsistency rate of 0.09% (out of 3,208,556 aligned reads) and the lowest suboptimal alignment rate of 1.2e − 06 (Fig. 6). Bowtie2 (“all” mode to report all alignments), Strobealign (“all”), and LAST also displayed a relatively low inconsistency rate of 0.31% (out of 3,209,652 aligned reads for Bowtie “all”), 2.40% (out of 3,210,658 aligned reads for Strobealign “all”), and 3.25% (out of 3,211,829 aligned reads for LAST). However, these came with a high cost of 73.5%, 53.4%, and 6.5%, respectively, of reads having suboptimal alignments. Other aligners exhibited 562–579-fold higher inconsistency rates of 52.0% (of 3,215,249 aligned reads for Minimap2 “all”), 51.9% (of 3,223,873 aligned reads for BWA “all”), 51.4% (of 3,210,436 aligned reads for Strobealign “default”), 52.1% (of 3,209,422 aligned reads for Bowtie2 “default”), 52.0% (of 3,215,249 aligned reads for Minimap2 “default”), and 52.0% (of 3,223,843 aligned reads for BWA “default”). Additionally, these aligners reported 4–133-fold higher suboptimal alignment rates than X-Mapper, at 1.6e − 04 (Minimap2 “all”), 1.6e − 04 (BWA “all”), 3.9e − 05 (Strobealign “default”), 5.6e − 06 (Bowtie2 “default”), 1.6e − 04 (Minimap2 “default”), and 6.5e − 06 (BWA “default”). We tested these aligners under four different penalty settings (default settings of Bowtie2, Minimap2, BWA, and X-Mapper) and consistently found that X-Mapper provided the most consistent alignments with the fewest suboptimal alignments. These observations indicate that X-Mapper is more consistent and more easily interpretable when aligning samples to complex references, such as microbial metagenomes. If users are interested in suboptimal alignments, that can be controlled via a separate parameter in X-Mapper (–max-penalty-span), which we did not test here.

### What makes X-Mapper more consistent?

To identify causes of alignment inconsistency, we also tested X-Mapper with ungapped x-mers and fixed k-mer sizes. We found that X-Mapper with ungapped x-mers showed a slightly higher inconsistency rate of 0.15% (of 3,208,056 aligned reads) than X-Mapper, which was similar to the inconsistency rates observed for Bowtie2 (“all”) (Fig. 6). We found that a fixed k-mer size (X-Mapper 16–24 mers) showed a higher inconsistency rate of 3.25–3.28% (of 3,197,876 to 3,211,829 aligned reads) than X-Mapper, which was similar to the inconsistency rates observed for LAST and Strobealign (“all”) (Fig. 6). Short k-mers, which can effectively avoid mutations but struggle to differentiate variant-dense regions in the reference genome, are more likely to yield too many matches for each k-mer when dealing with highly repetitive regions. This results in high inconsistency when aligning to a complex reference compared to a simple reference. Conversely, always using long k-mers is not ideal because they can be inefficient if the target strain is not well represented in the reference genome. This highlights the importance of using gapped x-mers, for aligning complex reference of diverse microbial species.

### Biological relevance of alignment consistency

To demonstrate the biological relevance of alignment consistency, we summarized the percentage of reads aligned to their own assembly, denoted “Assembly1,” or combinations of reference genomes that include Assembly1. Our hypothesis is that a good aligner should align reads from the WGS data back to their own assemblies as much as possible in a complex reference. We found that all aligners were able to align most of the WGS reads (3.2 million). However, these reads were assigned to different combinations of reference genomes by different aligners (Additional file 1: Fig. S7a).

Reads aligning to genome sets that contain the Assembly1 (A1, highlighted in purple) are likely true positive alignments. Reads aligning to genome sets that do not include A1 (labeled in blue) are likely false positive alignments. Except for X-Mapper, LAST, Strobealign (“all”), and Bowtie2 (“all”), the remaining aligners reported high false positive rates of 52.9–53.0%, indicating that other *B. fragilis* strains co-exist with the target *B. fragilis* strain when aligned to this complex reference (Additional file 1: Fig. S7a). We also tested another sample with the target *B. fragilis* strain mixed only with other *Bacteroides* species that are not *B. fragilis*. The remaining aligners also showed high false positive rates, with 30.0% of reads aligning to only the non-target *Bacteroides* species.

X-Mapper, along with LAST, Strobealign (“all”), and Bowtie2 (“all”), retrieved 95.6–98.9% of reads aligning back to A1 (Additional file 1: Fig. S7a). However, X-Mapper aligned a substantially higher proportion of reads uniquely to A1 (26%) compared to 5% (LAST), 12% (Strobealign “all”), and 5% (Bowtie2 “all”). This suggests that A1 is more competitive during alignment when using X-Mapper compared to the other three aligners.

To evaluate the specificity of each aligner’s alignments, we calculated the effective abundance of each genome as the effective average read depth that maps to a genome, which we expect to be 1 for A1 and 0 for all the other genomes. For each read, we determined the set of genomes to which this read was reported as aligned [19], filtered that set to genomes having ≥ 5% coverage, and added a read weight of 1 divided evenly among genomes in that set. X-Mapper retrieved 49.9% A1 abundance after normalization, while LAST, Strobealign (“all”), and Bowtie2 (“all”) retrieved 22.6%, 35.4%, and 23.5% A1 abundance, respectively (Additional file 1: Fig. S7b). Instead, the other three aligners mis-assigned higher abundances (10.8–15.5%) to non-target *B. fragilis* strains compared to X-Mapper (7.4–11.7%). This indicates that when aligning to the complex reference, the higher alignment consistency of X-Mapper results in more accurate taxonomy assignment and a more efficient abundance retrieval rate for the target strain.

### Speed of X-Mapper

We found that X-Mapper is competitive in speed compared to other aligners when tested on the same computer (30 GB RAM, 1–30 threads, and 3.00 GHz CPU speed) for reference indexing and read alignment (Additional file 1: Fig. S8). For read alignment, we found that X-Mapper (52.07 s with 15 threads) was generally faster than Bowtie2 (“with seed mismatches,” 111.05 s with 15 threads), LAST (231.43 s with 15 threads), and BWA (65.55 s with 15 threads), but slower than Strobealign (34.92 s with 15 threads) and Minimap2 (43.54 s with 15 threads) when aligning a real human gut microbiome metagenome of 9.3 million 100 bp reads to a 99.0 Mbp reference genome set — a dataset used for accuracy evaluation (Fig. 4a). For a simulated human gut microbiome metagenome of 3.2 million 150 bp reads aligned to a 441.4 Mbp reference genome set — a dataset generated for consistency evaluation (Fig. 6) — X-Mapper (97.87 s with 15 threads) was generally faster than Bowtie2 (“with seed mismatches,” 202.57 s with 15 threads), LAST (99.64 s with 15 threads), and Minimap2 (748.63 s with 15 threads), but slower than Strobealign (18.55 s with 15 threads) and BWA (37.69 s with 15 threads).

For reference indexing, we found that X-Mapper and LAST demonstrate a higher efficiency of multithreading, reducing the time from 57.85 s with one thread to 7.21 s with 30 threads (X-Mapper), and from 37.4 s with one thread to 9.8 s with 30 threads (LAST), for the real metagenome. However, indexing was more time-consuming for Bowtie2 and BWA, taking 92.49 s and 41.39 s for the real metagenome (Bowtie2), and 489.93 s and 257.8 s for the simulated metagenome (BWA), which is 1.1- to 4.3-fold longer than the time spent on read alignment (with 30 threads). Since X-Mapper continues indexing the reference genome during alignment if longer gapped x-mers are required, it is difficult to estimate the exact time spent on each part. We included only the initial round of reference indexing in the run time for indexing, while all other time was considered part of read alignment.

In terms of memory usage, X-Mapper is more resource-intensive than other aligners due to its storage of gapped x-mers. For a 99.0 Mbp reference, X-Mapper typically requires 3 GB of RAM, compared to Strobealign of 1.2 GB, LAST of 0.6 GB, Bowtie2 of 0.4 GB, Minimap2 of 1.1 GB, and BWA of 3.8 GB. For a 441.4 Mbp reference, X-Mapper requires 15–20 GB of RAM, compared to Strobealign (“all”) of 5.9 GB, LAST of 2.5 GB, Bowtie2 (“all”) of 2.4 GB, Minimap2 (“all”) of 3.7 GB, and BWA of 4.2 GB. The runtime of X-Mapper does not significantly decrease with increased memory (Additional file 1: Fig. S9).

**Fig. 7:**
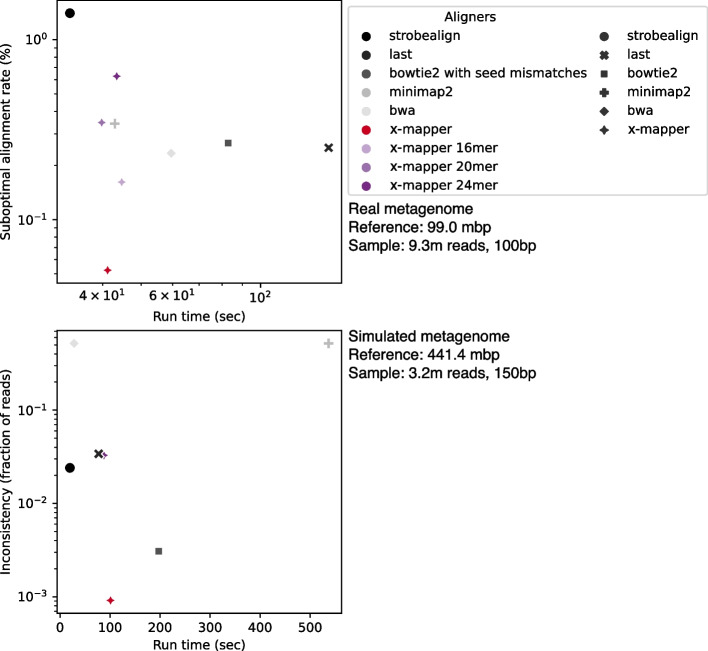
Run time of aligners tested on the same computer (30 GB RAM, 30 threads, 3.00 GHz CPU) for read alignment. Run time was measured for a human gut microbiome metagenome sample (the “real metagenome”) containing 9.3 million 100 bp reads aligned to its own 99.0 Mbp reference assembly, which was used for accuracy analysis (Fig. 4a); and a simulated human gut microbiome metagenome sample containing 3.2 million 150 bp reads aligned to a 441.4 Mbp reference dataset (the “simulated metagenome”), which was generated for consistency analysis (Fig. 6). The balance between speed and accuracy was compared across aligners, with accuracy measured by suboptimal alignments (for the “real metagenome”) and alignment inconsistency (for the “simulated metagenome”)

Alignment algorithms represent a balance between speed and accuracy. We evaluated this balance across aligners (Fig. 7), with accuracy measured by suboptimal alignments (for “the real metagenome”) and alignment inconsistency (for “the simulated metagenome”). We found that the relationship between alignment time (with 30 threads) and suboptimal alignment rates exhibits diminishing returns, where X-Mapper stands out as an outlier, offering the highest accuracy with competitive speed. As to alignment inconsistency, X-Mapper demonstrated 4-fold lower inconsistency than the second most consistent aligner, Bowtie2 (“all”), while requiring only half the alignment time. Compared to aligners with 37–38 times lower inconsistency (X-Mapper 16–24 mers, Strobealign “all,” and LAST), X-Mapper had 1–5-fold longer runtime. Given the increased availability of computing power and genomic data, we believe that an increase in memory and potentially some runtime in exchange for higher accuracy and consistency can be helpful for current research.

## Discussion

X-Mapper was designed to offer greater flexibility in filtering alignment results and to produce outputs that are more suitable for downstream analysis, such as filtered VCF files. In previous work [19], we found that alignment results can be incompletely summarized by tools like SAMtools [20] and BCFtools [21], which significantly affects the accuracy of downstream analyses, including genetic variant calling. Specifically, BCFtools (“bcftools call” command) frequently excludes indels in minimally overlapping paired-end reads. This exclusion of indels by BCFtools also leads to an underreporting of the major variant, which in turn elevates the frequency of minor variants, contributing to false positives in point mutation identification. Thus, X-Mapper includes specific settings (e.g., –max-penalty-span) that allows users to control the inclusion of suboptimal alignments within a specified penalty range or exclude them entirely.

To improve memory usage, we developed X-Mapper Next, which enables alignment to the human genome on a local PC with 16 GB of RAM, compared to X-Mapper’s memory usage of 48 GB. For bacterial genomes, X-Mapper Next reduces memory usage from 15–20 to 3 GB of RAM for a 441.4 Mbp reference and from 3 to 1 GB of RAM for a 99.0 Mbp reference.

X-Mapper Next shows comparable accuracy, consistency, and speed when tested with human gut microbiome sequencing data. The suboptimal alignment rate decreases from 0.05 to 0.04%, with the read alignment run time increasing from 41.17 to 59.50 s (30 threads, penalty setting #1, Additional file 1: Fig. S10). Meanwhile, the inconsistency rate drops from 0.09 to 0.08%, with the read alignment run time also decreasing from 100.99 to 84.79 s (30 threads, penalty setting #1, Additional file 1: Fig. S10). In addition, X-Mapper Next maintains an efficient reference indexing run time compared to X-Mapper, with 26.54 s versus 28.18 s for a 441.4 Mbp reference (30 threads) and 10.06 s versus 7.21 s for a 99.0 Mbp reference (30 threads). We are continuing to improve X-Mapper Next and perform more comprehensive testing on the human genome in future work.

A limitation of our work is that we have not significantly tested X-Mapper’s performance on long-read sequencing data, for which Minimap2 is specifically designed. This is currently being addressed in ongoing work. We have evaluated X-Mapper on human and diverse bacterial genomes, including both metagenome and transcriptome datasets, but have not yet tested it on non-microbial, non-human genomes. In addition, our evaluation using the human transcriptomic dataset may pose greater challenges for aligners designed primarily for genomic data alignment, as these aligners can behave differently when handling reads that span introns.

## Conclusions

This study introduced a new sequence alignment algorithm based on k-mers of dynamic sizes and gaps, which we call gapped x-mers. We use the gapped x-mer algorithm to develop a short-read alignment tool, which we call X-Mapper (https://github.com/mathjeff/Mapper). X-Mapper is a user-friendly application package, requiring only java. We found that X-Mapper displays the advantages of high accuracy, flexibility, and speed compared to existing aligners when applied to shotgun sequencing data from microbiome studies. Compared to Strobealign, LAST, Bowtie2, Minimap2, and BWA:

1. X-Mapper exhibits higher alignment accuracy than all other aligners, with a 5–33-fold lower suboptimal alignment rate when aligning a human gut microbiome metagenome and a 11–24-fold lower suboptimal alignment rate when aligning a human transcriptomic dataset. Ungapped x-mers and suboptimal k-mer sizes are major contributors to the wrong alignment sites for Strobealign (54.6%), Bowtie2 (56.6%), Minimap2 (67.5%), and BWA (59.2%) when aligning the human gut microbiome metagenome and for LAST (34.8%), Bowtie2 (32.7%), Minimap2 (25.9%), and BWA (22.6%) when aligning the human transcriptomic dataset.
2. X-Mapper reports a 3–579-fold lower alignment inconsistency compared to other aligners. Except for X-Mapper, LAST, Strobealign (“all”), and Bowtie2 (“all”), the remaining aligners report high false positive rates of 52.9–53.0% in identifying wrong strains and 30.0% in identifying wrong species. After normalizing the number of reads to the total number of genomes each read aligns to, X-Mapper retrieved 49.9% of abundance, while LAST, Strobealign (“all”), and Bowtie2 (“all”) retrieved 22.6%, 35.4%, and 23.5% A1 abundance, respectively.
3. When tested with human gut microbiome sequencing samples, X-Mapper showed competitive speed compared to other aligners. More importantly, it demonstrated higher accuracy, with a lower suboptimal alignment rate and lower alignment inconsistency compared to aligners of similar speed, indicating that X-Mapper achieves a more efficient balance between speed and accuracy.

In summary, this study demonstrates exploratory work that applied a gapped x-mer-based algorithm for short-read alignment, which created X-Mapper. X-Mapper was first tested against microbial genomes and shotgun sequencing. The accuracy, flexibility, and speed of X-Mapper suggest that in principle, this algorithm would potentially be useful to improve a variety of bioinformatics applications based on k-mers and x-mers — for example, similarity search (BLAST [3], Diamond [11]), taxonomy assignment in metagenomes (Kraken [4, 5]), multiple sequence alignment (MUSCLE [12], MAFFT [13]), and genome assembly (SPAdes [14], IDBA [15]). With the active application of sequencing in all biological fields, this advance can be a great improvement for many sequencing-based studies in the future.

## Methods

### X-Mapper usage

X-Mapper is a user-friendly application packaged as a jar (https://github.com/mathjeff/Mapper), which only requires java, and no special environment or additional runtime dependencies. Furthermore, X-Mapper can directly generate vcf files without requiring downstream tools such as BCFtools or SAMtools.

### X-Mapper algorithm

1. Build x-mers for reference.

X-Mapper starts by building a pyramid of x-mers to represent the reference (Fig. 2). The important properties of this process are (1) the pyramid construction process is determined solely by the sequence base pairs in the pyramid. If the same process is applied to the same sequence in the reference and to a portion of a query, the same pyramid will be produced. (2) Every base pair is covered by an x-mer in each level of the pyramid (different size of x-mers), except for x-mers that have become too large and would extend past the ends of the sequence. (3) The number of x-mers in each level of the pyramid usually decreases exponentially, which usually keeps the total number of x-mers in the pyramid less than about 4 times the length of the sequence.

First, X-Mapper builds an x-mer of length 1 bp for each position in the reference. To build the next level, it first visits each x-mer and chooses based on the x-mer’s contents whether that x-mer will request to merge with the x-mer to its left or to its right. Each x-mer of length 1 either requests to merge left or right, and encourages its descendants to request to merge left or right, for 4 combinations in total. Each of the 4 possible x-mers of length 1 (A, C, G, T) is assigned a different combination of these directions. For x-mers formed from merging two other x-mers, each x-mer usually identifies the parent with the larger hashcode and requests to merge left or right based on its recommendation. For the other parent, the child x-mer passes its recommendation along about which direction its descendants should merge with.

Next, X-Mapper visits every pair of adjacent x-mers, and if either x-mer in the pair requests to merge with the other, then X-Mapper performs a merge and adds the resulting x-mer into the next level of x-mers. Notice that for every x-mer that is not at the edge of the pyramid, it is guaranteed to produce a child x-mer in the following level of the pyramid. Also notice that if x-mers make independent, uniformly random decisions about which directions they request to merge in, then the probability of any particular child x-mer to exist is the probability that either parent requested its existence, which is approximately ¾ (= 1 − ½*½). This design generally causes the number of x-mers at each level to decrease exponentially as a function of the level number.

If X-Mapper sees an ambiguous base pair such as N, X-Mapper expands this into the relevant possibilities among A, C, G, and T. When multiple ambiguous x-mers merge, X-Mapper evaluates all of the possible combinations as long as the number of ambiguous base pairs is less than a limit (default setting is 3).

To reduce memory usage needed to index the reference genome, X-Mapper chooses hash functions and merge directions in a symmetric way such that the existence of an x-mer with a certain hashcode ensures the existence of the reverse complement x-mer with the same hashcode. In practice, this is done by temporarily keeping track of two hashcodes and two sets of merge direction requests and resolving them arbitrarily when needed (selecting those of the x-mer with higher hashcode).

2) Expand gapped x-mers.

When X-Mapper encounters an x-mer that seems interesting enough (long enough), X-Mapper first expands it into a gapped x-mer unless this is disabled via --no-gapmers. X-Mapper adds a gap of length equal to approximately half of the length of the x-mer, followed by a k-mer of approximately the same length. Each x-mer extends its gap to the left if it plans to next merge to the right, and extends its gap to the right if it plans to merge to the left. Each x-mer is then replaced with a new x-mer that ignores the contents of its gap.

3) Save x-mers into hashtable.

As X-Mapper generates x-mers, it assigns a hashcode to each x-mer based on its contents/sequence. The hashcode and the number of base pairs in the x-mer are then used together to identify a bin in the hashtable, and the position of the x-mer is added into the bin if there are not already too many x-mers in this bin (see the following section). This allows subsequent lookups to quickly identify all of the positions of an x-mer based solely on its hashcode and number of base pairs used.

If many x-mers are in a bin, then every subsequent lookup for the corresponding hashcode will yield that many matches and can cause alignments to be slow. To keep alignment fast, when X-Mapper is saving x-mers into the hashtable, it imposes a limit on the maximum number of x-mers that may be saved into any particular hash bin. Because discovering a match in a longer x-mer is both more unusual and more valuable than in a shorter x-mer, X-Mapper allows a different limit on the number of x-mers per bin as a function of the length of the x-mer, i.e., usually equal to the number of base pairs used by each x-mer in that bin.

Note that because the maximum number of positions in the reference genome saved of any small x-mer is also small, the total amount of space spent storing small x-mers is small, and the majority of space is spent storing medium-sized x-mers.

4) Build x-mers for queries and align queries using the x-mer pyramid.

After having built a database of x-mers describing the reference genome, X-Mapper visits each query separately and begins to build a similar pyramid for each. X-Mapper starts at the x-mer in the first position of the first row of the pyramid for the query and does a lookup in its x-mer database to see how many positions that x-mer can be found. If the number of positions that an x-mer finds exceeds the maximum number allowed, then X-Mapper proceeds to the next position of the next row of the pyramid. Otherwise, X-Mapper saves that x-mer and its corresponding alignment offsets (positions of the query in the reference) and returns to the next position in the previous level of the pyramid. This process is repeated to form a path of x-mers through the pyramid, which dynamically adjusts the level of the pyramid and therefore also adjusts the length of the x-mers based on the number of matches of each in the reference. If gapped x-mers were enabled when indexing the reference, gapped x-mers are used when analyzing the query, too.

5) Optimistic check for single best alignment.

X-Mapper follows this x-mer path until one reference offset is found having more x-mer matches than any other, and at least two x-mer matches in total. If this process yields exactly one position, X-Mapper checks it first and attempts to determine (using the nonexistence of x-mer matches at other offsets) that it is the best alignment. If X-Mapper is unable to determine that this optimistic alignment is optimal (which can often be demonstrated via the nonexistence of enough x-mers matching at other offsets), then X-Mapper continues to follow this x-mer path.

6) Visit offsets in priority order.

If X-Mapper is unable to demonstrate that the optimistic best alignment is optimal, it continues to follow the x-mer path and looks for new matching offsets. For each discovered offset, X-Mapper attempts an alignment at that position. Positions that are discovered earlier entail fewer initial mismatched x-mers, and are considered higher priority and are checked before later positions [22].

X-Mapper generally disregards offsets until two supporting x-mers are found for the same position because positions having only one supporting x-mer are likely to yield poor alignments and may even be the product of hash collisions where the hashtable has stored different x-mers with different contents into the same bin.

The process of checking candidate offsets continues until X-Mapper finds an alignment and can demonstrate that no other candidate offset can provide a lower penalty (due to the nonexistence of x-mer matches), or X-Mapper can demonstrate that no candidate offset can provide a satisfactory penalty, or until the x-mer path reaches the end of the query.

7) Refine bound on alignment penalty.

Once X-Mapper finds an offset that seems worthwhile to check for a possible alignment, X-Mapper attempts to compute a bound on the maximum possible alignment penalty at this position, to improve the performance of the subsequent search for indels.

First an ungapped alignment is checked, and if its penalty is less than the penalty of a single gap (indel), then that alignment is used. Next, X-Mapper identifies the region around the candidate alignment and extracts short pieces (length approximately log(query length * 3 + 1) / log(4) + 1) from it. X-Mapper also extracts short pieces from the query and searches for the maximum number of nonoverlapping, nonmatching pieces that can be detected. Each such nonmatching piece is expected to contribute at least one mutation to the final alignment. If this penalty is higher than what X-Mapper is interested in, the offset is rejected. Lastly, X-Mapper computes the maximum length of extensions that can exist in an optimal alignment at this offset based on the maximum interesting penalty and the minimum number of mismatched pieces. If the maximum possible indel length is 0, an ungapped alignments is reported.

8) Split query and join alignments.

If X-Mapper is unable to determine the nonexistence of indels in the best alignment at the offset, X-Mapper next splits the query into several pieces and for each one, separately refines the bound on the alignment penalty (step 7) and searches for indels (step 9). If the best alignments for two adjacent pieces of the query are adjacent, those alignments are re-joined into one contiguous alignment. If neighboring pieces yield best alignments that are nonadjacent, X-Mapper proceeds to thoroughly check for indels in the next step.

9) Check for indels.

The last step in X-Mapper is to identify the optimal alignment overlapping a given offset via dynamic programming.

The first step in the indel identification process is to generate a lazy Needleman-Wunsch grid [23], which can determine the penalty of an individual alignment. Each possible alignment is modeled as a path through this two-dimensional grid, where a movement in the x-direction represents the alignment of a base pair in the query, and a movement in the y-direction represents the alignment of a base pair in the reference. This allows each box in this grid to be aware of the minimum penalty required to reach that box from the start of the grid.

The next step in the indel identification process is to search this Needleman-Wunsch grid for the path having minimum penalty. This is done using the A* search algorithm [18], plus pruning any branches that have exceeded the maximum indel extension length calculated previously for this offset.

When the A* search completes, it produces a path that can be transformed into an alignment, and the alignment process for that particular query offset is done. Other possible offsets might still need to be checked as previously described, depending on the exact alignment penalties.

### Evaluating alignment accuracy of X-Mapper in microbial samples with various complexities

To evaluate the alignment accuracy, we tested X-Mapper (version 1.1.0-beta09), Bowtie2 [24] (version 2.5.1, default and -N 1), BWA [25] (version 0.7.17-r1188), Minimap2 [2] (version 2.26-r1175), Strobealign [26] (version 0.13.0), and LAST [7] (version 1584) to align the same sequence dataset to the same reference. The datasets included (1) a human gut microbiome metagenome (NCBI accession SAMN10410254) mapping to its own assembly, representing a complex reference; (2) a human transcriptomic dataset (NCBI accession DRR163384) mapping to a complete human genome (GCF_009914755.1); and (3) a WGS sample of *B. fragilis* (NCBI accession SAMN11846534) mapping to its own assembly (SAMN11943505), to different strains of the same species (SAMN11943558 isolated from the same person; SAMN11943586 and SAMN11943574 isolated from different people), and to genomes generated from one WGS assembly (SAMN11943558) with various inserted mutation densities (5e − 4, 2e − 2, 3e − 2, 4e − 2, and 5e − 2); and to other species, including *B. caccae* (NCBI accession SAMN11943406), *B. stercoris* (NCBI accession SAMN11943815), *B. ovatus* (SAMN11943669), and *B. xylanisolvens* (SAMN11944338).

For a fair comparison, we tested all aligners under the same penalty settings:

1. The default setting of Bowtie2 (penalty setting #1):
  X-Mapper: “--max-penalty 0.60 --snp-penalty 6 --new-indel-penalty 5 --extend-indel-penalty 3 --ambiguity-penalty 1”
  Bowtie2: “--mp 6,6 --rfg 5,3 --rdg 5,3 --np 1 --ignore-quals”
  Minimap2: “-ax sr -B 4 -O 3 -E 3 -A 2 --score-N 1” (“-A 2” as default)
  BWA: “-B 5 -O 4 -E 3 -A 1 -L 100” (“-A 1” as default). The “-L 100” setting was applied to prevent soft clipping. Here, we kept the default settings of “-A 2” for Minimap2 and “-A 1” for BWA, while adjusting the “-B” and “-O” scores such that “-A” score + “-B” score equaled the total point mutation penalty and “-A” score + “-O” score equaled the total gap open penalty.
  Strobealign: “-B 6 -O 5 -E 3 -A 0 -L 1000.” The -L 1000 parameter was applied to prevent excessive soft clipping, for example, preventing a 100 bp soft clip with only 1 match. We also tested configurations with “-A” set to 0 (“-B 6 -O 5 -E 3”), 1 (“-B 5 -O 4 -E 3”), and 2 (“-B 4 -O 3 -E 3”), which produced similar results (the highest suboptimal alignment rates). Here, the setting “-A 0” was used because the default setting, “-A 2,” occasionally triggered an error in samtools during “samtools view -F 4,256” processing (even though Strobealign itself did not report any errors).
  LAST: “last-train -q 4 -a 3 -b 3 -r 2 -X 1 -Q1” followed by “lastal -q 4 -a 3 -b 3 -r 2 -X 1 -T1 -p reads.train.” The “T1” setting (overlap alignment) was applied to prevent excessive soft clipping.
2. The default setting of BWA (penalty setting #2):
  X-Mapper: “--max-penalty 0.50 --snp-penalty 5 --new-indel-penalty 6 --extend-indel-penalty 1”
  Bowtie2: “--mp 5,5 --rfg 6,1 --rdg 6,1 --np 1 --ignore-quals”
  Minimap2: “-ax sr -B 3 -O 4 -E 1 -A 2 --score-N 1”
  BWA: “-B 4 -O 5 -E 1 -A 1 -L 100”
  Strobealign: “-B 5 -O 6 -E 1 -A 0 -L 1000”
  LAST: “last-train -q 3 -a 4 -b 1 -r 2 -X 1 -Q1” followed by “lastal -q 3 -a 4 -b 1 -r 2 -X 1 -T1 -p reads.train”
3. The default setting of X-Mapper (penalty setting #3):
  X-Mapper: “--max-penalty 1.00 --snp-penalty 10 --new-indel-penalty 20 --extend-indel-penalty 5 --ambiguity-penalty 1”
  Bowtie2: “--mp 10,10 --rfg 20,5 --rdg 20,5 --np 1 --ignore-quals”
  Minimap2: “-ax sr -B 8 -O 18 -E 5 -A 2 --score-N 1”
  BWA: “-B 9 -O 19 -E 5 -A 1 -L 100”
  Strobealign: “-B 10 -O 20 -E 5 -A 0 -L 1000”
  LAST: “last-train -q 8 -a 18 -b 5 -r 2 -X 1 -Q1” followed by “lastal -q 8 -a 18 -b 5 -r 2 -X 1 -T1 -p reads.train”
4. The default setting of Minimap2 (penalty setting #4):
  X-Mapper: “--max-penalty 0.60 --snp-penalty 6 --new-indel-penalty 4 --extend-indel-penalty 2 --ambiguity-penalty 1”
  Bowtie2: “--mp 6,6 --rfg 4,2 --rdg 4,2 --np 1 --ignore-quals”
  Minimap2: “-ax sr -B 4 -O 2 -E 2 -A 2 --score-N 1”
  BWA: “-B 5 -O 3 -E 2 -A 1 -L 100”
  Strobealign: “-B 6 -O 4 -E 2 -A 0 -L 1000”
  LAST: “last-train -q 4 -a 2 -b 2 -r 2 -X 1 -Q1” followed by “lastal -q 4 -a 2 -b 2 -r 2 -X 1 -T1 -p reads.train”

Here, we tested single-end alignment because different tools have varying algorithms for computing pair-end penalties, which could result in different preferences for the best alignments. We used a stand-alone script to re-compute the penalty of an alignment, using the CIGAR string and position to confirm the number of matches, mismatches, ambiguous matches, gap opens, and gap extensions.

Regarding soft clips, we consider them not important for short-read alignment, as both X-Mapper and Bowtie2 emphasize the contiguity of alignment. However, Minimap2 and BWA often report soft clips in the middle of a reference contig under their default settings. We found that changing the soft clip penalty works for BWA (-L), but not for Minimap2 (-z, even when trying various scores/penalties from 100 to 1 billion). For a fair comparison for Minimap2, we removed reads reported with soft clips in the middle of the contigs by Minimap2, which accounted for 0.2–1% of all reads (Figs. 4 and 5). We also conducted another analysis that kept all reads and treated soft clips in the middle of the contigs as indels. The results showed the same trend, with X-Mapper reporting the lowest suboptimal alignment rates (Fig. 4) than the other aligners.

The default maximum allowed penalty of X-Mapper is 10 point mutations per 100 bp match (“–max-penalty”), allowing twice the divergence of a species-level ANI of 95% [17]. For references with more than 10% dissimilarity to the sample, we recommend using a more representative reference or adjusting the “–max-penalty” setting to allow for more mutations. Thus, alignments from other aligners exceeding 10% dissimilarity were excluded from downstream analysis.

The optimal alignments of a read were identified as those with the lowest penalty across all alignments reported by the aligners. Alignments with higher penalties were considered suboptimal. We used suboptimal alignment rates to represent alignment accuracy, computed as the number of reads reported with suboptimal alignments by an aligner divided by the total number of reads in the input sequence dataset. To determine the potential cause of suboptimal alignments, we tested X-Mapper with fixed k-mer sizes (--block-length k) and ungapped x-mers (--no-gapmers) to see if a suboptimal k-mer size could replicate the suboptimal alignments. We confirmed the potential cause by adjusting the k-mer size for Minimap2 (-k and -w during library indexing), BWA (-k minimum seed length), and Bowtie2 (--seedlen).

### Evaluating alignment consistency of X-Mapper in samples with various complexities

We tested the ability of aligners to report consistent alignments for the same read when aligning it to a single reference genome versus complex reference genomes representing diverse microbial species. Specifically, we compared the alignment of one *B. fragilis* WGS dataset (NCBI accession SAMN11943556) to its own assembly (Assembly1) with the alignment of this WGS dataset to a collection of 88 human gut microbiome genomes (Additional file 2: Table S1). The genome collection included (1) the *B. fragilis* WGS assembly (Assembly1); (2) 5 other *B. fragilis* references representing different strains; (3) 55 *Bacteroides* species that are not *B. fragilis*; and (4) 27 species that are not *Bacteroides*.

For each read, we compared its alignments to the complex reference with those to the simple reference. An alignment was characterized as consistent if: (1) the optimal alignments to the simple reference were also reported when aligned to the complex reference, or (2) the complex reference produced better alignments (with lower penalties) than those in the simple reference. We then computed inconsistency as the percentage of reads that did not have consistent alignments when aligned to the complex reference compared to the simple reference.

For Strobealign, LAST, Bowtie2 (“-N 1”), Minimap2, and BWA, we used the total penalty of a pair of paired-end reads to identify their optimal alignments. X-Mapper selected the optimal alignments based on Combined Alignment Scores (cs), which consider both the alignment penalties of paired-end reads and the spacing penalties between them. Therefore, we used cs to determine the optimal alignments of a pair of paired-end reads for X-Mapper.

We ran all aligners using four different penalty settings (as described above). For X-Mapper, we adjusted spacing penalty for paired-end reads proportionally based on the point mutation penalty. We disabled secondary suboptimal alignments (–max-penalty-span 0) to avoid reporting suboptimal alignments.

### Short read speed test

For a fair comparison in terms of run time, all aligners (X-Mapper, Minimap2, Bowtie2 “-N 1,” BWA, Strobealign, and LAST) were tested on the same sequence dataset aligning to the same reference genome under their default penalty settings. We used two testing datasets: (1) a human gut microbiome metagenome (NCBI accession SAMN10410254, 100 bp, 9.3 million reads) aligning to its own assembly (99.0 mbp) and (2) a simulated human gut microbiome metagenome (NCBI accession SAMN11943558, 1.6 million reads, 150 bp) aligning to a reference collection of 88 genomes (441.4 mbp, Additional file 2: Table S1). For the simulated human gut microbiome metagenome dataset, Bowtie2, Minimap2, BWA, and Strobealign were set to output multiple, if not all, alignments (-a or -N 100). All samples were run on the same computer with 30 GB RAM, 1–30 threads, and a 3.00 GHz CPU. By default, Java allocates 25% of system memory. In this analysis, Java was allocated a maximum of 30 GB of RAM to run X-Mapper (java -Xms30g -Xmx30g). The same process, from reference indexing to read alignments, was tested on all aligners.

### Code and data availability

All data is publicly available on DOI: https://doi.org/10.6084/m9.figshare.25976434 [27]. All code is publicly available on https://github.com/mathjeff/Mapper [10] (X-Mapper DOI: https://doi.org/10.5281/zenodo.14258690 [28] and X-Mapper Next DOI: https://doi.org/10.5281/zenodo.14258790 [29]) and https://github.com/caozhichongchong/Mapper_eva (DOI: https://doi.org/10.5281/zenodo.14252193 [30]). Code was tested on python v3.7 and jupyter notebook v5.7.1.

## Supplementary Information

Additional file 1: Supplementary figures. Figs. S1–S10

Additional file 2: Table S1 NCBI genome identifiers for the 88 human gut microbiome genomes used in the consistency analysis

## Data Availability

All data is publicly available on DOI: https://doi.org/10.6084/m9.figshare.25976434 [27]. All datasets are publicly available, including NCBI accessions SAMN11943556, SAMN11846534, SAMN11943558, SAMN11943505, SAMN11943586, SAMN11943574, SAMN11943406, SAMN11943815, SAMN11943669, and SAMN11944338 [31], NCBI accession SAMN10410254 [32], and NCBI accession DRR163384 [33]. All code is publicly available on https://github.com/mathjeff/Mapper [10] (X-Mapper DOI: http://doi.org/10.5281/zenodo.14258690 [28] and X-Mapper Next DOI: http://doi.org/10.5281/zenodo.14258790 [29] licensed under the MIT license) and https://github.com/caozhichongchong/Mapper_eva (DOI: http://doi.org/10.5281/zenodo.14252193 [30]). Code was tested on python v3.7 and jupyter notebook v5.7.1.

## References

[1] Lloyd-Price J, Mahurkar A, Rahnavard G, Crabtree J, Orvis J, Hall AB, et al. Strains, functions and dynamics in the expanded Human Microbiome Project. Nature. 2017;550:61–6. 28953883 10.1038/nature23889 PMC5831082

[2] Li H. Minimap2: pairwise alignment for nucleotide sequences. Bioinformatics. 2018;34:3094–100. 29750242 10.1093/bioinformatics/bty191 PMC6137996

[3] Camacho C, Coulouris G, Avagyan V, Ma N, Papadopoulos J, Bealer K, et al. BLAST+: architecture and applications. BMC Bioinformatics. 2009;10:421. 20003500 10.1186/1471-2105-10-421 PMC2803857

[4] Wood DE, Salzberg SL. Kraken: ultrafast metagenomic sequence classification using exact alignments. Genome Biol. 2014;15:R46. 24580807 10.1186/gb-2014-15-3-r46 PMC4053813

[5] Wood DE, Lu J, Langmead B. Improved metagenomic analysis with Kraken 2. Genome Biol. 2019;20:257. 31779668 10.1186/s13059-019-1891-0 PMC6883579

[6] Chikhi R, Medvedev P. Informed and automated k-mer size selection for genome assembly. Bioinformatics. 2014;30:31–7. 23732276 10.1093/bioinformatics/btt310

[7] Kiełbasa SM, Wan R, Sato K, Horton P, Frith MC. Adaptive seeds tame genomic sequence comparison. Genome Res. 2011;21:487–93. 21209072 10.1101/gr.113985.110 PMC3044862

[8] Ma B, Tromp J, Li M. PatternHunter: faster and more sensitive homology search. Bioinformatics. 2002;18:440–5. 11934743 10.1093/bioinformatics/18.3.440

[9] David M, Dzamba M, Lister D, Ilie L, Brudno M. SHRiMP2: sensitive yet practical short read mapping. Bioinformatics. 2011;27:1011–2. 21278192 10.1093/bioinformatics/btr046

[10] Gaston J, Zhang A-N. X-Mapper [Internet]. Github; 2024. Available from: https://github.com/mathjeff/Mapper

[11] Buchfink B, Xie C, Huson DH. Fast and sensitive protein alignment using DIAMOND. Nat Methods. 2015;12:59. 25402007 10.1038/nmeth.3176

[12] Edgar RC. MUSCLE: multiple sequence alignment with high accuracy and high throughput. Nucleic Acids Res. 2004;32:1792–7. 15034147 10.1093/nar/gkh340 PMC390337

[13] Katoh K, Standley DM. MAFFT multiple sequence alignment software version 7: improvements in performance and usability. Mol Biol Evol. 2013;30:772–80. 23329690 10.1093/molbev/mst010 PMC3603318

[14] Bankevich A, Nurk S, Antipov D, Gurevich AA, Dvorkin M, Kulikov AS, et al. SPAdes: a new genome assembly algorithm and its applications to single-cell sequencing. J Comput Biol. 2012;19:455–77. 22506599 10.1089/cmb.2012.0021 PMC3342519

[15] Peng Y, Leung HC, Yiu S-M, Chin FY. Meta-IDBA: a de novo assembler for metagenomic data. Bioinformatics. 2011;27:i94-101. 21685107 10.1093/bioinformatics/btr216 PMC3117360

[16] Poyet M, Groussin M, Gibbons S, Avila-Pacheco J, Jiang X, Kearney S, et al. A library of human gut bacterial isolates paired with longitudinal multiomics data enables mechanistic microbiome research. Nat Med. 2019;25:1442–52. 31477907 10.1038/s41591-019-0559-3

[17] Jain C, Rodriguez-R LM, Phillippy AM, Konstantinidis KT, Aluru S. High-throughput ANI analysis of 90K prokaryotic genomes reveals clear species boundaries. bioRxiv. 2017;225342. 10.1038/s41467-018-07641-9 PMC6269478 30504855

[18] Hart PE, Nilsson NJ, Raphael B. A formal basis for the heuristic determination of minimum cost paths. IEEE Transact Syst Sci Cybernet. 1968;4:100–7.

[19] Gaston JM, Alm EJ, Zhang A-N. Fast and accurate variant identification tool for sequencing-based studies. BMC Biol. 2024;22:90. 38644496 10.1186/s12915-024-01891-4 PMC11034086

[20] Li H, Handsaker B, Wysoker A, Fennell T, Ruan J, Homer N, et al. The sequence alignment/map format and SAMtools. Bioinformatics. 2009;25:2078–9. 19505943 10.1093/bioinformatics/btp352 PMC2723002

[21] Danecek P, Bonfield JK, Liddle J, Marshall J, Ohan V, Pollard MO, et al. Twelve years of SAMtools and BCFtools. Gigascience. 2021;10:giab008. 33590861 10.1093/gigascience/giab008 PMC7931819

[22] Liao Y, Smyth GK, Shi W. The Subread aligner: fast, accurate and scalable read mapping by seed-and-vote. Nucleic Acids Res. 2013;41:e108–e108. 23558742 10.1093/nar/gkt214 PMC3664803

[23] Needleman SB, Wunsch CD. A general method applicable to the search for similarities in the amino acid sequence of two proteins. J Mol Biol. 1970;48:443–53. 5420325 10.1016/0022-2836(70)90057-4

[24] Langmead B, Salzberg SL. Fast gapped-read alignment with Bowtie 2. Nat Methods. 2012;9:357. 22388286 10.1038/nmeth.1923 PMC3322381

[25] Li H, Durbin R. Fast and accurate short read alignment with Burrows-Wheeler transform. Bioinformatics. 2009;25:1754–60. 19451168 10.1093/bioinformatics/btp324 PMC2705234

[26] Sahlin K. Strobealign: flexible seed size enables ultra-fast and accurate read alignment. Genome Biol. 2022;23:260. 36522758 10.1186/s13059-022-02831-7 PMC9753264

[27] Gaston J, Zhang A-N. Supplementary data for “X-Mapper: fast and accurate sequence alignment via gapped x-mers” [Internet]. 2024. Available from: 10.6084/m9.figshare.25976434

[28] Gaston J, Zhang A-N. X-Mapper: 1.1.0-beta09 [Internet]. Github; 2024. 10.5281/zenodo.14258690

[29] Gaston J, Zhang A-N. X-Mapper: 1.1.0-beta11 [Internet]. Github; 2024. 10.5281/zenodo.14258790

[30] Gaston J, Zhang A-N. Code for paper “X-Mapper: fast and accurate sequence alignment via x-mers” [Internet]. Github; 2024. Available from: 10.5281/zenodo.14252193

[31] Poyet M, Groussin M, Gibbons S, Avila-Pacheco J, Jiang X, Kearney S, et al. BIO-ML: the Broad Institute-OpenBiome Microbiome Library enables mechanistic studies with isolates, genomes, and longitudinal metagenomics and metabolomic data [Internet]. 2019. Available from: https://www.ncbi.nlm.nih.gov/bioproject/544527

[32] Poyet M, Alm E. Human gut metagenome raw sequence reads [Internet]. 2018. Available from: https://www.ncbi.nlm.nih.gov/bioproject/503484

[33] Ritsumeikan University. EphA2 antisense RNA regulates proliferation and migration of human breast cancer cells by modulating EphA2 mRNA, and protein expression levels [Internet]. 2024. Available from: https://www.ncbi.nlm.nih.gov/bioproject/PRJDB7825

