# Author Correction: X-Mapper: fast and accurate sequence alignment via gapped x-mers

**DOI:** 10.1186/s13059-025-03481-1

**Published:** 2025-01-27

**Authors:** Jeffry M. Gaston, Eric J. Alm, An-Ni Zhang

**Affiliations:** 1 Google, Cambridge, MA USA; 2 Department of Biological Engineering, Massachusetts Institute of Technology, Cambridge, MA USA; 3 School of Biological Sciences, Nanyang Technological University, Singapore, Singapore

**Correction** **:** **Genome Biol 26, 15 (2025)**

**https://doi.org/10.1186/s13059-024-03473-7**

Following publication of the original article [1], the authors identified that one of the headings in the results section is incorrect.

The incorrect heading is: Alignment accuracy of X‑Mapper in samples with various ties

The correct heading is: Alignment accuracy of X‑Mapper in samples with various complexities

The original article [1] has been updated.
