# Supplementary material for: X-Mapper: fast and accurate sequence alignment via gapped x-mers
Source: Genome Biol. 2025 Jan 22;26:15. doi: 10.1186/s13059-024-03473-7 (PMC11755882; doi:10.1186/s13059-024-03473-7)
Supplement: Supplementary file 1 — Additional file 1: Supplementary figures. Figs. S1–S10 [file 13059_2024_3473_MOESM1_ESM.pdf]

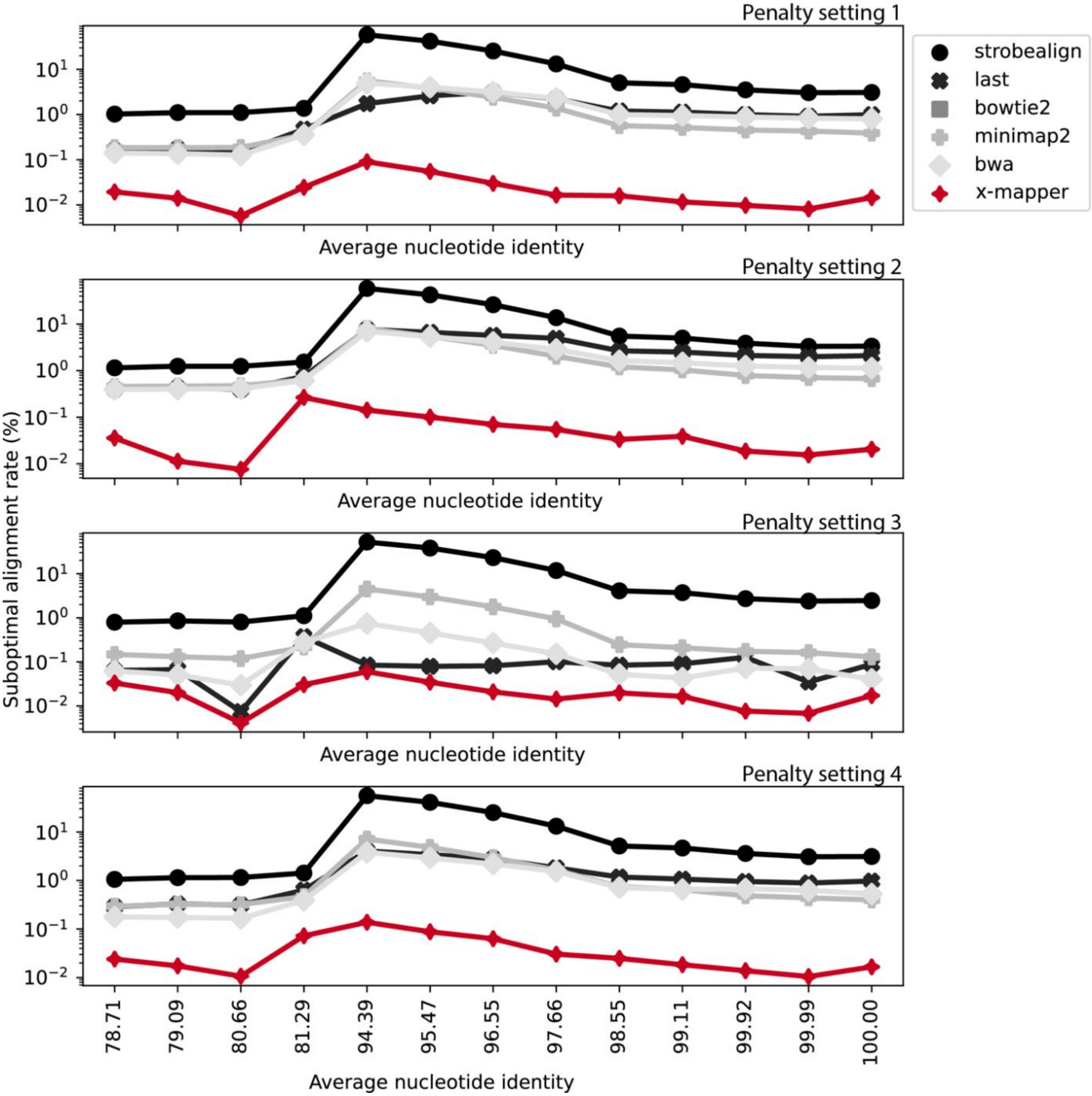

Fig S1. X-Mapper exhibits higher alignment accuracy with a lower suboptimal alignment rate (percentage of reads reported with suboptimal alignments) than other aligners, when applied to reference genomes with various ANI compared to the sequencing sample.

Lowest penalty: 25 (optimal)
Aligner 1 penalty: 30 (suboptimal)

1. Long k-mers unmatched

Aligners	Penalty
X-Mapper (12mer)	25
X-Mapper (16mer)	25
X-Mapper (20mer)	30
X-Mapper (24mer)	30

2. Short k-mers unmatched

Aligners	Penalty
X-Mapper (12mer)	30
X-Mapper (16mer)	30
X-Mapper (20mer)	30
X-Mapper (24mer)	25

3. Short and long k-mers unmatched

Aligners	Penalty
X-Mapper (12mer)	30
X-Mapper (16mer)	30
X-Mapper (20mer)	25
X-Mapper (24mer)	30

4. Ungapped x-mer unmatched

Aligners	Penalty
X-Mapper	25
X-Mapper (no gapped x-mer)	30

5. Soft clips missing

Optimal alignment	Aligner 1
Soft clip 1	No soft clip 1

6. Indels missing

Optimal alignment	Aligner 1
Indel 1	No indel 1

7. Misassigned soft clips

Optimal alignment	Aligner 1
No soft clip 2	Soft clip 2

Fig S2. A flowchart illustrating the process of assigning potential causes for suboptimal alignments. An example is provided: the optimal alignment yields the lowest penalty of 25, while a suboptimal alignment reported by Aligner 1 has a higher penalty of 30. Steps 1–7 are followed to identify the potential cause of this suboptimal alignment. Once a cause is identified, the process is terminated.

```
Aligned query: GTAAGGTTTTGGTGGAGCGTACTGAGAATATGCAATGGATGCCGCGTTCCAACCGCTATTATTATACCCGTACAGGGGTGGATGGCAGACAGCTGAT
Difference   :             !            !                 !     !     !                 !  !           !
Aligned ref  : GTAAGGTTTTGGCGGAGCGTACTGAAAATATGCAATGGATGCCTCGTTCTAACCGTTATTATTATACCCGTACGGGAGTGGATGGCAGGCAGCTGAT
```

Not mapping	Mapping
X-Mapper (ungapped x-mers)<br>X-Mapper (12mer)<br>X-Mapper (22mer)<br>X-Mapper (24mer)	X-Mapper<br>X-Mapper (14mer)<br>X-Mapper (16mer)<br>X-Mapper (18mer)<br>X-Mapper (20mer)
Strobealign<br>Strobealign (15mer)<br>Strobealign(16mer)	Strobealign (10mer)<br>Strobealign(14mer)
	LAST
Bowtie2<br>Bowtie2 (18mer)	Bowtie2 (14mer)<br>Bowtie2 (with seed mismatches)
Minimap2<br>Minimap2 (16mer)	Minimap2 (12mer)<br>Minimap2 (14mer)
BWA<br>BWA (10mer)<br>BWA (12mer)<br>BWA (14mer)	BWA (18mer)

Fig S3. An example where only X-Mapper was able to report an alignment, while the other aligners failed under their default k-mer size settings, demonstrating that unmatched long k-mers might be the potential cause. Alignments reported by aligners using default settings were highlighted with underlines.

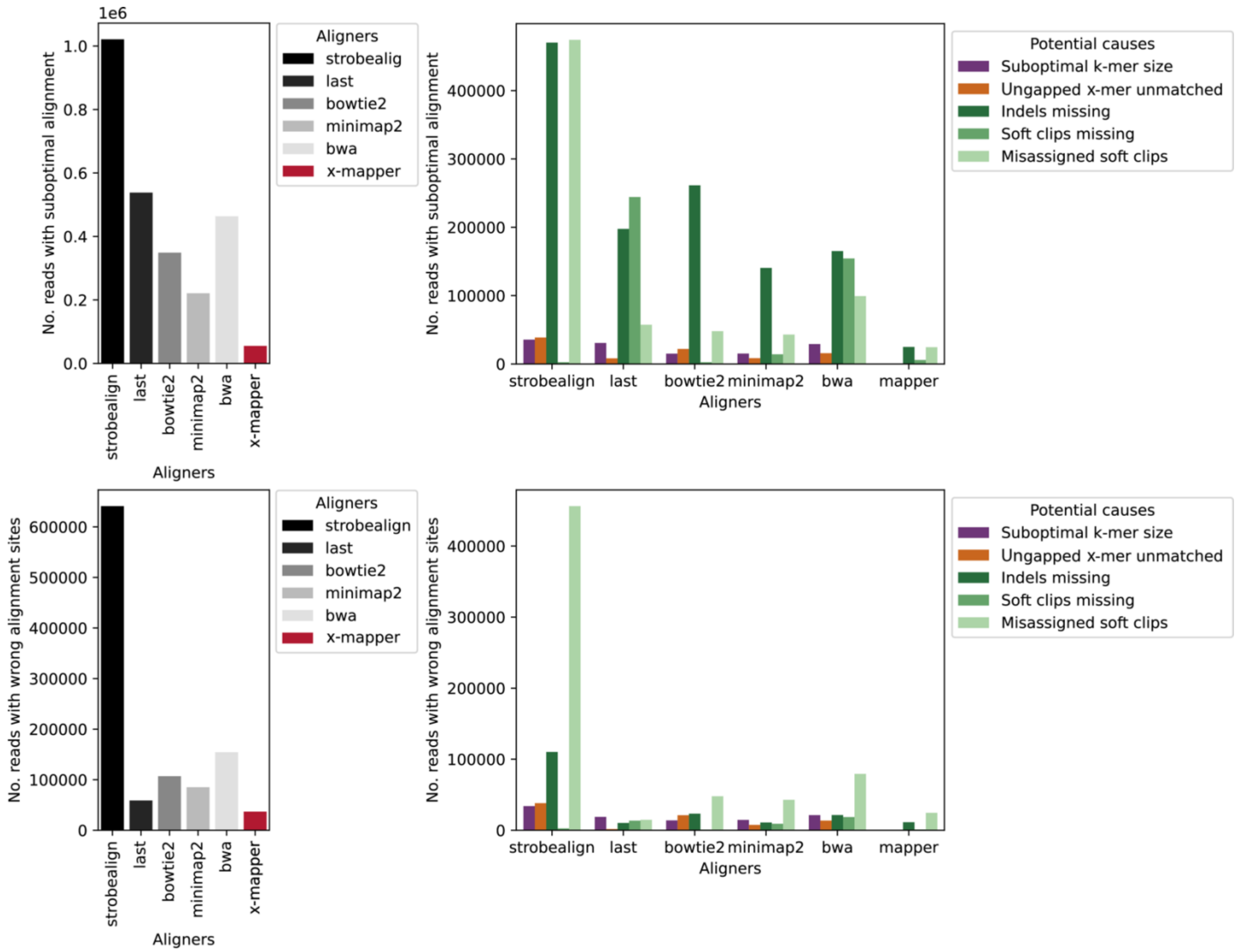

Fig S4. Potential causes of suboptimal alignments when aligning a human transcriptomic dataset. Suboptimal alignments were classified into four types: reads failed to align, aligned to the wrong sites, aligned to close sites (within 100 bp), or aligned to the same sites but with a higher (suboptimal) penalty score compared to the optimal alignments. Reads that failed to align or aligned to the wrong sites were considered more serious suboptimal alignment types due to their greater potential to impact downstream analysis. The Bowtie2 configuration with seed mismatches was tested in this analysis.

Penalty settings

SNP	Indel start	Indel extension	Ambiguity
6	5	3	1

X-Mapper

```
Optimal alignment - POS 170744
 Aligned query: ATACAGAGGATACCATTTGC--ATGACATCCTCTGTATTTACCGAA…
 Difference   :            !!            !!
 Aligned ref  : ATA-----GATGTCA--TGCAAATGGTATCCTCTGTATTTACCGAA…
Penalty = 66
```

SNP	Indel start	Indel extension	Ambiguity
6*4	5*3	3*9	1*0

Bowtie2

```
Suboptimal alignment - POS 170753
 Aligned query: ATACAGAGGATACCATTTGCATGACATCCTCTGTATTTACCGAA…
 Difference   :   !                    !!
  Aligned ref : ATGCA---------A-----ATGGTATCCTCTGTATTTACCGAA…
Penalty = 70
```

SNP	Indel start	Indel extension	Ambiguity
6*3	5*2	3*14	1*0

Minimap2
BWA

```
Suboptimal alignment - POS 170764
 Aligned query: ATACAGAGGATACCATTTGCATGACATCCTCTGTATTTACCGAA…
 Difference   :
  Aligned ref : -------------------------ATCCTCTGTATTTACCGAA…
Penalty = 80
```

SNP	Indel start	Indel extension	Ambiguity
6*0	5*1	3*25	1*0

Strobealign

```
Suboptimal alignment - POS 170702
 Aligned query: ATACAGAGGATACCATTTGCATGACATCCTCTGTATTTACCGAA…
 Difference   :    !!! !!!!!!!!!!!!!!!!!!!!! !!! !!!!!! !! !
  Aligned ref : ATATTTATCGGCTATCCAAACGACGTAACGGGGATCGATCGTAT…
Penalty = 216
```

SNP	Indel start	Indel extension	Ambiguity
6*36	5*0	3*0	1*0

Last **No alignment**

Fig S5. An example where aligners identified approximate sites in the reference genome but reported different arrangements of matches, resulting in higher penalties or suboptimal alignments. Mismatches, including point mutations, soft clips, and indels, are highlighted in red.

Penalty settings

SNP	Indel start	Indel extension	Ambiguity
6	5	3	1

Minimap2

```
Optimal alignment - POS 24316
 Aligned query: ATCATATTCATATCCATTGTTTTATTCCTTTCTTTTAGATGTTCAAACCAGAGAAACACATAAATGCCATTGCCATCAGACCTACAGTGATAAACGTGATG
Difference    :                                                                               !            SSSSS
 Aligned ref  : ATCATATTCATATCCATTGTTTTATTCCTTTCTTTTAGATGTTCAAACCAGAGAAACACATAAATGCCATTGCCATCAAACCTACAGTGATGAAAGSSSSS
Penalty = 16
```

SNP	Indel start	Indel extension	Ambiguity
6*1	5*0	3*0	1*10

Bowtie2

```
Suboptimal alignment - POS 24316
 Aligned query: ATCATATTCATATCCATTGTTTTATTCCTTTCTTTTAGATGTTCAAACCAGAGAAACACATAAATGCCATTGCCATCAGACCTACAGTGATAAACGTGATG
Difference    :                                                                               !
 Aligned ref  : ATCATATTCATATCCATTGTTTTATTCCTTTCTTTTAGATGTTCAAACCAGAGAAACACATAAATGCCATTGCCATCAAACCTACA---------GTGATG
Penalty = 38
```

SNP	Indel start	Indel extension	Ambiguity
6*1	5*1	3*9	1*0

X-Mapper

```
Suboptimal alignment - POS 24316
Aligned query:  ATCATATTCATATCCATTGTTTTATTCCTTTCTTTTAGATGTTCAAACCAGAGAAACACATAAATGCCATTGCCATCAGACCTACAGTGATAAACGTGATG
 Difference   :                                                                               !            !  !
 Aligned ref  : ATCATATTCATATCCATTGTTTTATTCCTTTCTTTTAGATGTTCAAACCAGAGAAACACATAAATGCCATTGCCATCAAACCTACAGTGATGAAAGSSSSS
Penalty = 23
```

SNP	Indel start	Indel extension	Ambiguity
6*3	5*0	3*0	1*5

Strobealign

```
Suboptimal alignment - POS 24265
 Aligned query: ATCATATTCATATCCATTGTTTTATTCCTTTCTTTTAGATGTTCAAACCAGAGAAACACATAAATGCCATTGCCATCAGACCTACAGTGATAAACGTGATG
 Difference   :   ! ! !!! !!!!!  !!!!!!!!!!!!!!! !! !! !!!!!!!!!! ! !! !!!!!!! !!!!!!! !!!  !!!!!!!!!!!!   !!! !!!!!!
 Aligned ref  : ATAACAACAAGCAGGATGACAACCACGAGGAATACTCCAATGCTTGCTAATATCATATTCATATCCATTGTTTTATTCCTTTCTTTTAGATGTTCAAACCA
Penalty = 480
```

SNP	Indel start	Indel extension	Ambiguity
6*80	5*0	3*0	1*0

Last

```
Suboptimal alignment - POS 24316
 Aligned query: ATCATATTCATATCCATTGTTTTATTCCTTTCTTTTAGATGTTCAAACCAGAGAAACACATAAATGCCATTGCCATCAGACCTACAGTGATAAACGTGATG
Difference    :                                                                               !            !
 Aligned ref  : ATCATATTCATATCCATTGTTTTATTCCTTTCTTTTAGATGTTCAAACCAGAGAAACACATAAATGCCATTGCCATCAAACCTACAGTGATGAA----A-G
Penalty = 37
```

SNP	Indel start	Indel extension	Ambiguity
6*2	5*2	3*5	1*0

BWA

```
Suboptimal alignment - POS 24316
 Aligned query: ATCATATTCATATCCATTGTTTTATTCCTTTCTTTTAGATGTTCAAACCAGAGAAACACATAAATGCCATTGCCATCAGACCTACAGTGAT-AAA-CGTGATG
 Difference   :                                                                               !
 Aligned ref  : ATCATATTCATATCCATTGTTTTATTCCTTTCTTTTAGATGTTCAAACCAGAGAAACACATAAATGCCATTGCCATCAAACCTACAGTGATGAAAGSSSSSSS
Penalty = 29
```

SNP	Indel start	Indel extension	Ambiguity
6*1	5*2	3*2	1*7

Fig S6. An example where aligners (LAST, Bowtie2, Minimap2, BWA, and X-Mapper) reported alignments of one read to the same site in the reference (POS 24,316) while disagreeing on the arrangement of matches. Mismatches, including point mutations, soft clips, and indels, are highlighted in red. Soft clips are labeled as “S.”

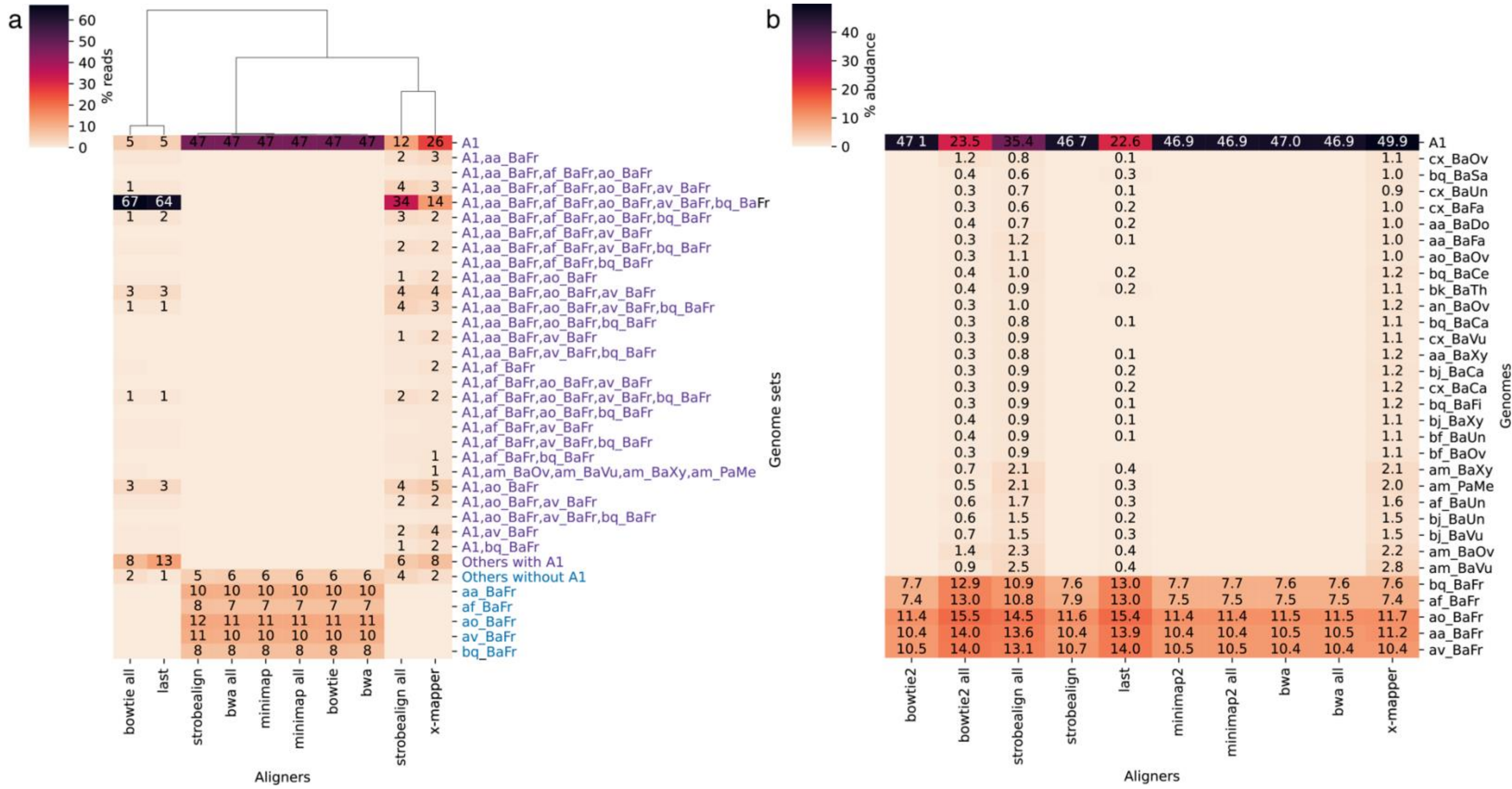

Fig S7. Percentage of reads aligning to different combinations of reference genomes (a) and effective abundance of each reference genome covered by reads (b). (a) Values were labeled if >= 0.1%. Genome sets containing the Assembly1 of the original WGS data are labeled in purple (likely true positives). Genome sets not containing the Assembly1 of the original WGS data are labeled in blue (likely false positives). (b) Values were labeled if >= 1%. Genomes with coverage < 5% reported by all aligners were discarded.

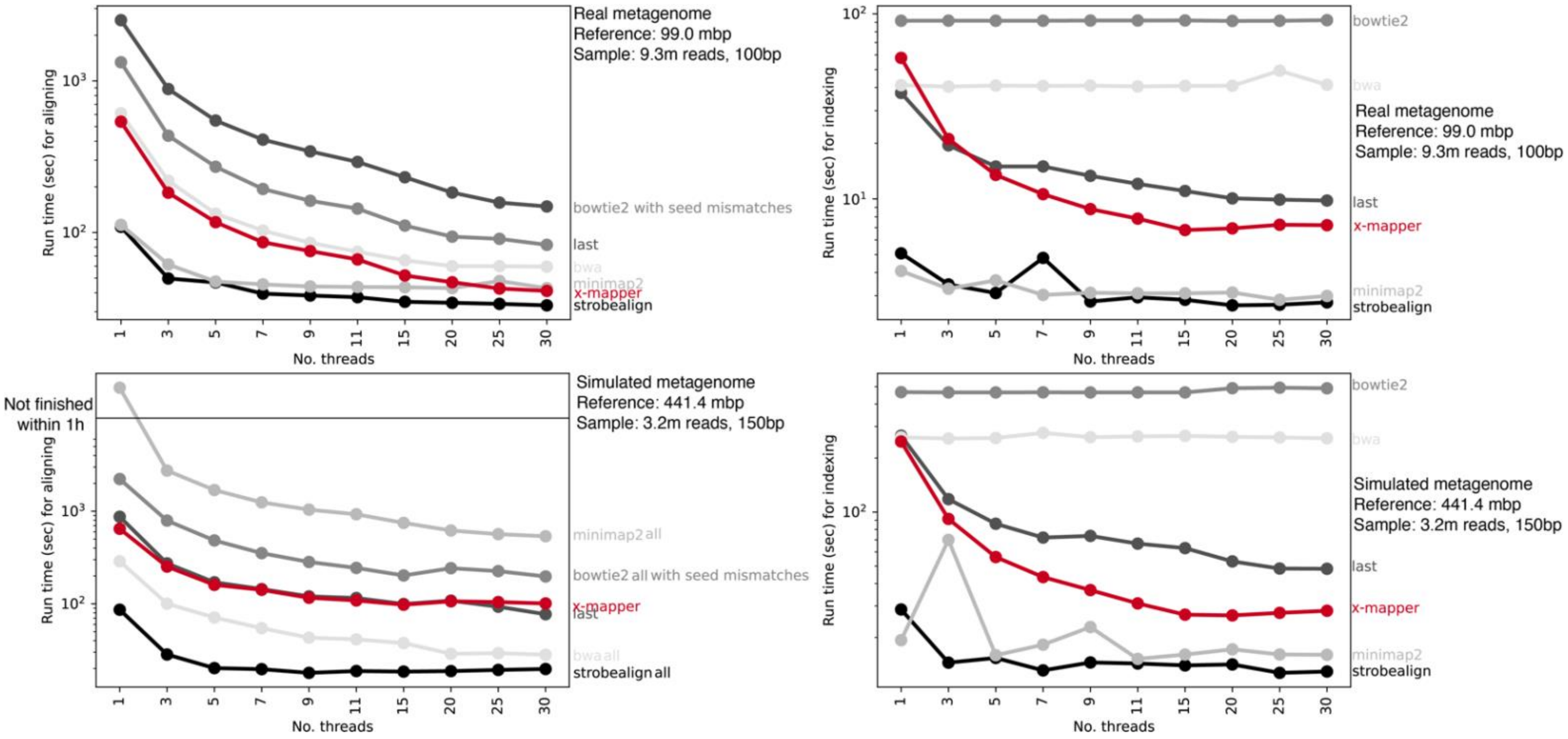

Fig S8. Run time of aligners tested on the same computer (30 GB RAM, 1-30 threads, 3.00GHz CPU) for reference indexing and read alignment. Run time was measured for a human gut microbiome metagenome sample (“the real metagenome”) containing 9.3 million 100 bp reads aligned to its own 99.0 Mbp reference assembly, and a simulated human gut microbiome metagenome sample containing 3.2 million 150 bp reads aligned to a 441.4 Mbp reference dataset (“the simulated metagenome”).

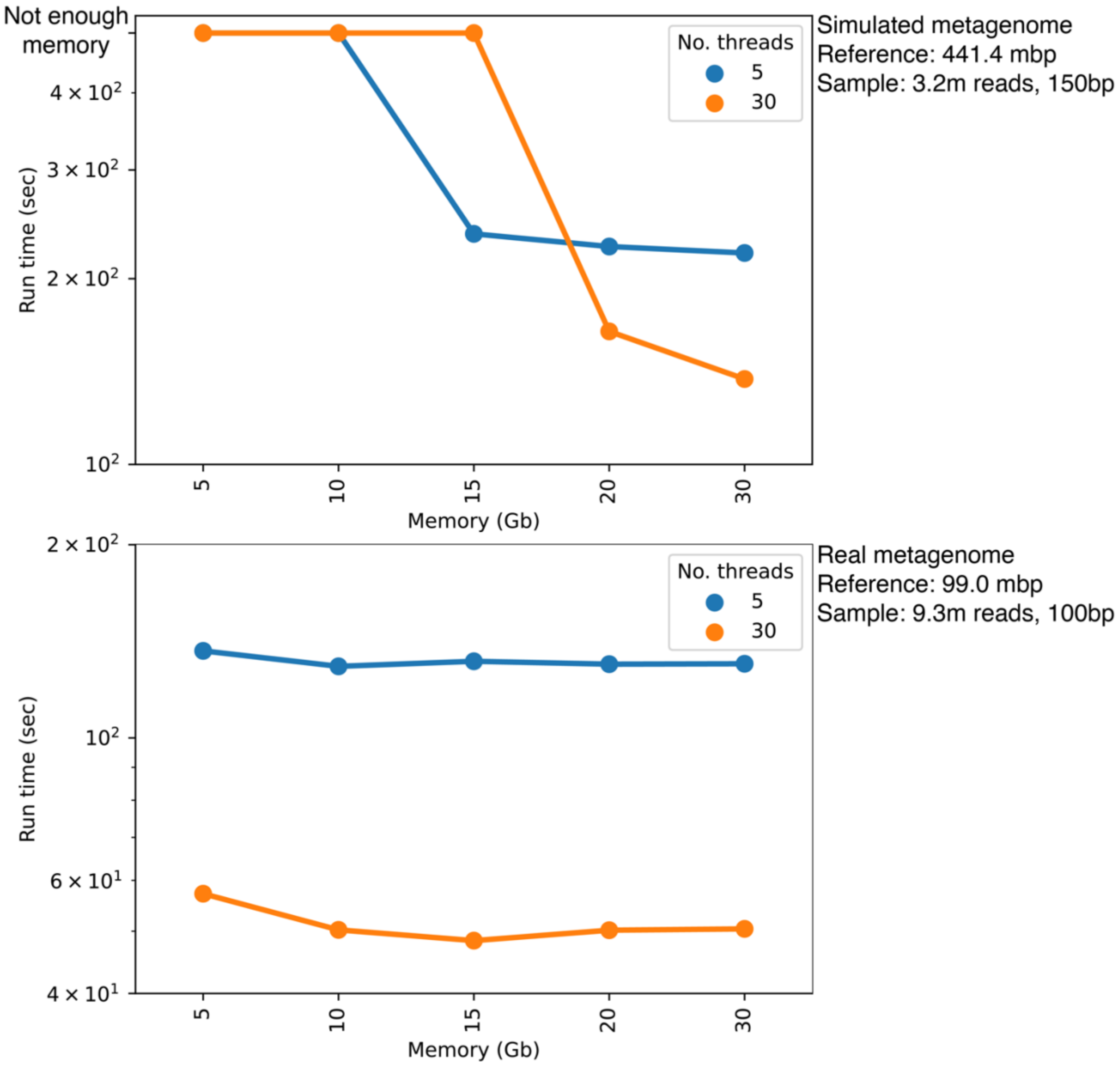

Fig S9. Run time of aligners tested on the same computer (5-30 GB RAM, 5 and 30 threads, 3.00GHz CPU) for reference indexing and read alignment. Run time was measured for a human gut microbiome metagenome sample ("the real metagenome") containing 9.3 million 100 bp reads aligned to its own 99.0 Mbp reference assembly, and a simulated human gut microbiome metagenome sample containing 3.2 million 150 bp reads aligned to a 441.4 Mbp reference dataset ("the simulated metagenome"). In this analysis, Java was allocated a maximum of N GB of RAM to run X-Mapper (java -XmsNg -XmxNg).

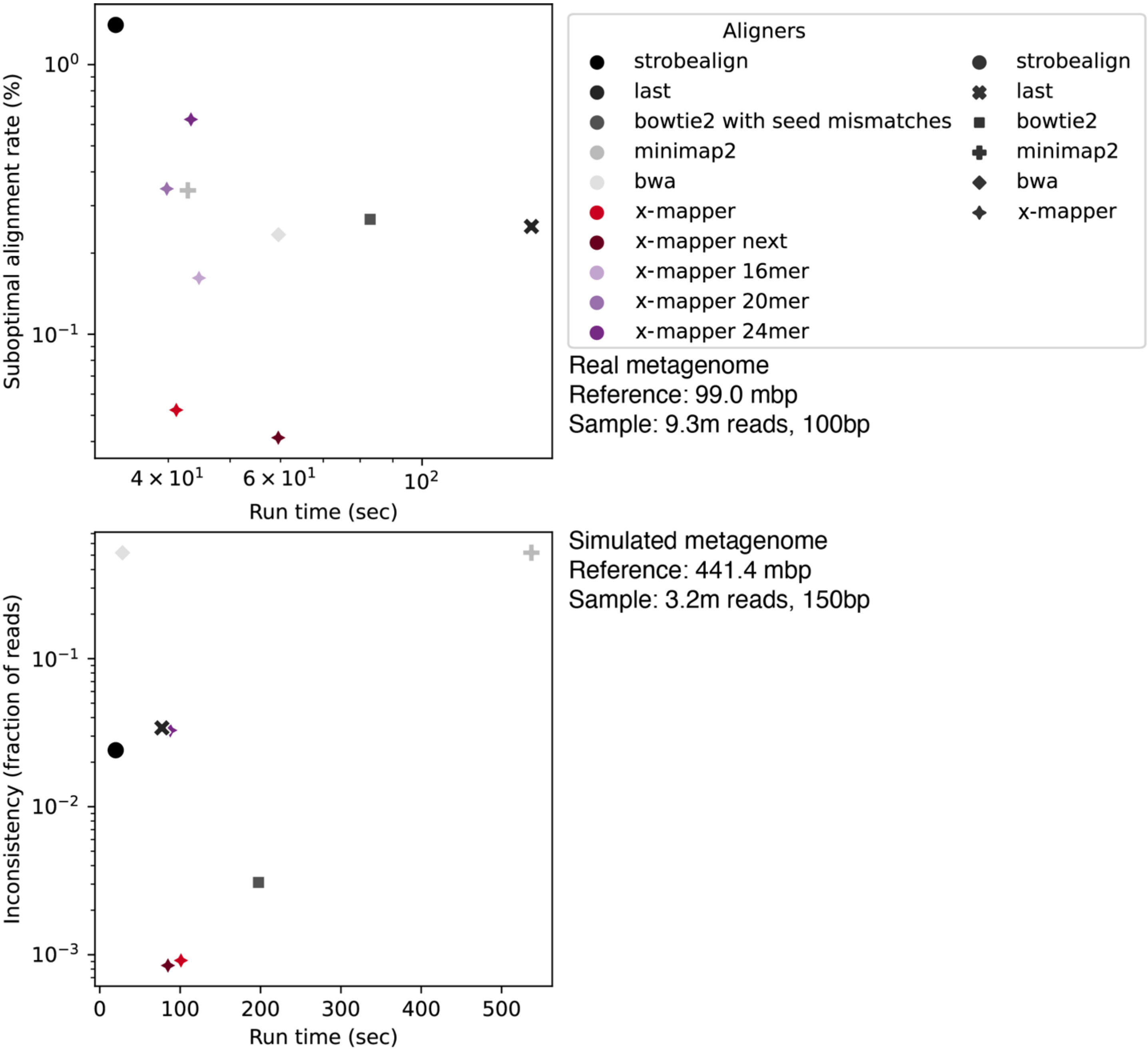

Fig S10. Run time of X-Mapper Next and X-Mapper compared to other aligners tested on the same computer (30 GB RAM, 30 threads, 3.00GHz CPU) for read alignment. Run time was measured for a human gut microbiome metagenome sample (the “real metagenome”) containing 9.3 million 100 bp reads aligned to its own 99.0 Mbp reference assembly, which was used for accuracy analysis (Fig. 4a); and a simulated human gut microbiome metagenome sample containing 3.2 million 150 bp reads aligned to a 441.4 Mbp reference dataset (the “simulated metagenome”), which was

generated for consistency analysis (Fig. 6). The balance between speed and accuracy was compared across aligners, with accuracy measured by suboptimal alignments (for the “real metagenome”) and alignment inconsistency (for the “simulated metagenome”).
